# A second monoclinic polymorph of 3β-chloro­cholest-5-ene

**DOI:** 10.1107/S1600536810015722

**Published:** 2010-05-08

**Authors:** Mohd. Shaheen Khan, Othman Sulaiman, Issam Ahmed Mohamed, Chin Sing Yeap, Hoong-Kun Fun

**Affiliations:** a Bio-resource, Paper and Coatings Technology Division, School of Industrial Technology, Universiti Sains Malaysia, 11800 USM, Penang, Malaysia; b X-ray Crystallography Unit, School of Physics, Universiti Sains Malaysia, 11800 USM, Penang, Malaysia

## Abstract

The title compound, C_27_H_45_Cl, is a second monoclinic polymorph which crystallizes in the space group *P*2_1_ with four crystallographically independent mol­ecules in the asymmetric unit. The structure was previously reported [Bernal *et al.* (1940 ▶). *Philos. Trans. R. Soc. London Ser. B*, **239**, 135–182; Vani & Vijayan (1979 ▶). *Mol. Cryst. Liq. Cryst.* **51**, 253–264], also in the space group *P*2_1_, but with two unique mol­ecules in the asymmetric unit. As in the previously reported structures, rings *A* and *C* in the mol­ecule adopt chair conformations with half-chair conformations for rings *B* and *D*. The ring junctions *B*–*C* and *C*–*D* are *trans*, whereas the junction *A*–*B* is quasi-*trans*. In the crystal structure, mol­ecules are arranged in a head-to-tail fashion along *a* and are stacked along the *b* axis.

## Related literature

For general background to steroid compounds, see: Doorenbos & Wu (1968 ▶); Green *et al.* (1978 ▶); Clinton & Manso (1961 ▶); Rajnikant *et al.* (2006 ▶). For the structures of the other polymorphs of the title compound, see: Bernal *et al.* (1940 ▶); Vani & Vijayan (1979 ▶). For ring conformations, see: Cremer & Pople (1975 ▶). For the melting point of the title compound, see: Baker & Squire (1948 ▶). For the stability of the temperature controller used in the data collection, see: Cosier & Glazer, (1986 ▶).
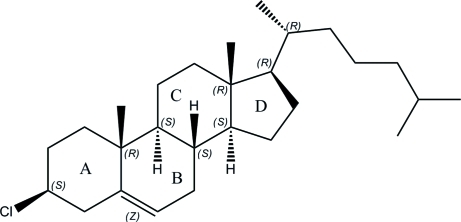


## Experimental

### 

#### Crystal data

C_27_H_45_Cl
*M* *_r_* = 405.08
Monoclinic, 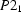

*a* = 21.208 (2) Å
*b* = 7.5302 (7) Å
*c* = 30.778 (3) Å
β = 91.453 (2)°
*V* = 4913.7 (8) Å^3^
*Z* = 8
Mo *K*α radiation
μ = 0.17 mm^−1^
*T* = 100 K
0.42 × 0.31 × 0.09 mm

#### Data collection

Bruker APEXII DUO CCD area-detector diffractometer
Absorption correction: multi-scan (*SADABS*; Bruker, 2009 ▶) *T*_min_ = 0.933, *T*_max_ = 0.985
78605 measured reflections
28604 independent reflections
18381 reflections with *I* > 2σ(*I*)
*R*_int_ = 0.067

#### Refinement

*R*[*F* ^2^ > 2σ(*F* ^2^)] = 0.057
*wR*(*F* ^2^) = 0.144
*S* = 0.98
28604 reflections
1017 parameters
1 restraint
H-atom parameters constrained
Δρ_max_ = 0.41 e Å^−3^
Δρ_min_ = −0.35 e Å^−3^
Absolute structure: Flack (1983 ▶), 13239 Friedel pairs
Flack parameter: −0.01 (4)

### 

Data collection: *APEX2* (Bruker, 2009 ▶); cell refinement: *SAINT* (Bruker, 2009 ▶); data reduction: *SAINT*; program(s) used to solve structure: *SHELXTL* (Sheldrick, 2008 ▶); program(s) used to refine structure: *SHELXTL*; molecular graphics: *SHELXTL*; software used to prepare material for publication: *SHELXTL* and *PLATON* (Spek, 2009 ▶).

## Supplementary Material

Crystal structure: contains datablocks global, I. DOI: 10.1107/S1600536810015722/sj2768sup1.cif

Structure factors: contains datablocks I. DOI: 10.1107/S1600536810015722/sj2768Isup2.hkl

Additional supplementary materials:  crystallographic information; 3D view; checkCIF report

## Figures and Tables

**Fig. 1.**
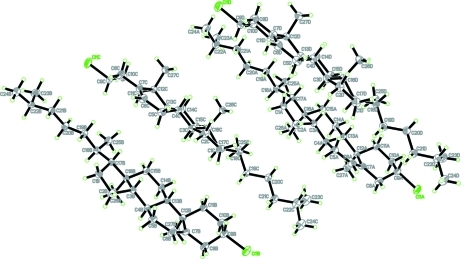
The asymmetric unit of (I), showing 50% probability displacement ellipsoids and the atom-numbering scheme.

**Fig. 2.**
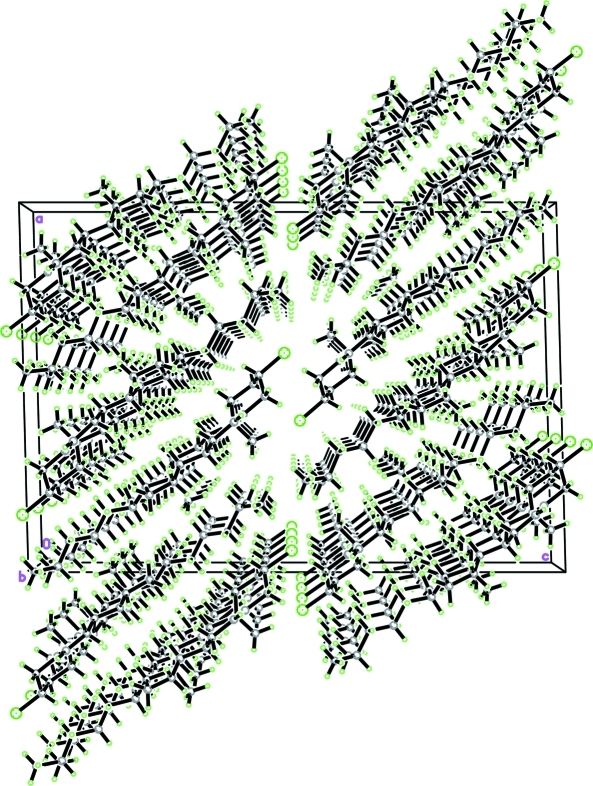
The crystal packing of (I) viewed along the b axis, showing the molecules stack along b axis.

## supplementary crystallographic information

### Comment

For the last seventy years the chemistry of steroids has provided one of the most interesting and thoroughly explored areas for organic chemists. The synthetic modification of naturally occurring steroids with the hope of improving pharmacological essentials has resulted in the preparation and discovery of a number of diverse pharmacologically active, potent, highly specific commercially important therapeutic agents (Doorenbos & Wu, 1968; Green *et al.*, 1978; Clinton & Manso, 1961). The cholesterol molecule in steroidal chemistry is well known as it comprises of four-ring structure of which three are six-membered cyclohexane rings and one is a five membered carbon ring (Rajnikant *et al.*, 2006). In the present work an attempt has been made to synthesize a derivative of the cholesterol molecule. Although the title compound (I) crystallized in the same monoclinic space group, *P*2_1_, as previously reported (Bernal *et al.*, 1940; Vani & Vijayan, 1979), the unit cell is different.

The asymmetric unit of the title compound (I) consists of four crystallographically independent molecules whereas the previously reported structures had only two unique molecules in the asymmetric unit of a different monoclinic unit cell. As with the previously reported structures, rings A and C adopt chair conformations with half chair conformations for rings B and D (Cremer & Pople, 1975). The ring junctions B–C and C–D are *trans* whereas the junction A–B is quasi *trans*. In the crystal strucutre, molecules are arrange head-to-tail down *a* axis and each molecule is stacked along the *b* axis (Fig. 2).

### Experimental

Freshly purified thionyl chloride (75 ml) was added gradually to cholesterol (100 g) at room temperature. A vigorous reaction ensued with the evolution of gaseous products. When the reaction slackened, the mixture was gently heated at the temperature of 325-335 K on a water bath for 1 h and then poured onto crushed ice-water mixture with stirring. The yellow solid thus obtained was filtered under suction and washed several times with ice-cooled water and air dried. Recrystallization of crude product from acetone gave (I) (94 g), m.p. 368-369 K (reported m.p. 369-370 K; Baker & Squire, 1948). It gave positive Beilstein test and a yellow colour with tetra-nitromethane in chloroform.

### Refinement

All H atoms were positioned geometrically and refined using a riding model, with C–H = 0.93–0.98 Å, *U*_iso_(H) = 1.2 or 1.5 *U*_eq_(C). The rotating group model was applied for the methyl groups. The same U_ij_ parameters were used for atom pairs C23C/C24C and C23A/C24A. A total of 13239 Friedel pairs were use to determine the absolute configuration.

### Crystal data

**Table:**

C_27_H_45_Cl	*F*(000) = 1792
*M**_r_* = 405.08	*D*_x_ = 1.095 Mg m^−^^3^
Monoclinic, *P*2_1_	Mo *K*α radiation, λ = 0.71073 Å
Hall symbol: P 2yb	Cell parameters from 9910 reflections
*a* = 21.208 (2) Å	θ = 2.7–29.9°
*b* = 7.5302 (7) Å	µ = 0.17 mm^−^^1^
*c* = 30.778 (3) Å	*T* = 100 K
β = 91.453 (2)°	Plate, colourless
*V* = 4913.7 (8) Å^3^	0.42 × 0.31 × 0.09 mm
*Z* = 8	

### Data collection

**Table:**

Bruker APEXII DUO CCD area-detector diffractometer	28604 independent reflections
Radiation source: fine-focus sealed tube	18381 reflections with *I* > 2σ(*I*)
graphite	*R*_int_ = 0.067
φ and ω scans	θ_max_ = 30.0°, θ_min_ = 1.0°
Absorption correction: multi-scan (*SADABS*; Bruker, 2009)	*h* = −29→29
*T*_min_ = 0.933, *T*_max_ = 0.985	*k* = −10→10
78605 measured reflections	*l* = −42→43

### Refinement

**Table:**

Refinement on *F*^2^	Secondary atom site location: difference Fourier map
Least-squares matrix: full	Hydrogen site location: inferred from neighbouring sites
*R*[*F*^2^ > 2σ(*F*^2^)] = 0.057	H-atom parameters constrained
*wR*(*F*^2^) = 0.144	*w* = 1/[σ^2^(*F*_o_^2^) + (0.0663*P*)^2^] where *P* = (*F*_o_^2^ + 2*F*_c_^2^)/3
*S* = 0.98	(Δ/σ)_max_ = 0.002
28604 reflections	Δρ_max_ = 0.41 e Å^−^^3^
1017 parameters	Δρ_min_ = −0.35 e Å^−^^3^
1 restraint	Absolute structure: Flack (1983), 13239 Friedel pairs
Primary atom site location: structure-invariant direct methods	Flack parameter: −0.01 (4)

### Special details

**Table:**

Experimental. The crystal was placed in the cold stream of an Oxford Cryosystems Cobra open-flow nitrogen cryostat (Cosier & Glazer, 1986) operating at 100.0 (1) K.
Geometry. All esds (except the esd in the dihedral angle between two l.s. planes) are estimated using the full covariance matrix. The cell esds are taken into account individually in the estimation of esds in distances, angles and torsion angles; correlations between esds in cell parameters are only used when they are defined by crystal symmetry. An approximate (isotropic) treatment of cell esds is used for estimating esds involving l.s. planes.
Refinement. Refinement of *F*^2^ against ALL reflections. The weighted *R*-factor wR and goodness of fit *S* are based on *F*^2^, conventional *R*-factors *R* are based on *F*, with *F* set to zero for negative *F*^2^. The threshold expression of *F*^2^ > σ(*F*^2^) is used only for calculating *R*-factors(gt) etc. and is not relevant to the choice of reflections for refinement. *R*-factors based on *F*^2^ are statistically about twice as large as those based on *F*, and *R*- factors based on ALL data will be even larger.

### Fractional atomic coordinates and isotropic or equivalent isotropic displacement parameters (Å^2^)

**Table:**

	*x*	*y*	*z*	*U*_iso_*/*U*_eq_	
Cl1A	1.08384 (3)	0.05358 (10)	0.491683 (19)	0.03717 (16)	
C1A	0.73950 (11)	0.2488 (3)	0.23594 (7)	0.0239 (5)	
H1AA	0.7553	0.3137	0.2113	0.029*	
H1AB	0.6942	0.2656	0.2368	0.029*	
C2A	0.77135 (11)	0.3165 (3)	0.27850 (7)	0.0264 (5)	
H2AA	0.7958	0.4231	0.2734	0.032*	
H2AB	0.7403	0.3415	0.3003	0.032*	
C3A	0.81402 (10)	0.1626 (3)	0.29261 (7)	0.0187 (4)	
H3AA	0.8508	0.1685	0.2740	0.022*	
C4A	0.84017 (10)	0.1599 (3)	0.33922 (7)	0.0197 (4)	
H4AA	0.8052	0.1440	0.3591	0.024*	
C5A	0.87390 (11)	0.3344 (3)	0.35018 (7)	0.0244 (5)	
H5AA	0.8978	0.3716	0.3253	0.029*	
H5AB	0.8426	0.4251	0.3556	0.029*	
C6A	0.91738 (11)	0.3206 (3)	0.38878 (7)	0.0274 (5)	
H6AA	0.9333	0.4258	0.4005	0.033*	
C7A	0.93539 (11)	0.1685 (3)	0.40787 (7)	0.0235 (5)	
C8A	0.97827 (12)	0.1723 (3)	0.44809 (7)	0.0302 (5)	
H8AA	0.9927	0.2928	0.4533	0.036*	
H8AB	0.9549	0.1342	0.4731	0.036*	
C9A	1.03468 (11)	0.0514 (4)	0.44247 (7)	0.0298 (5)	
H9AA	1.0595	0.0961	0.4184	0.036*	
C10A	1.01370 (11)	−0.1358 (3)	0.43209 (7)	0.0280 (5)	
H10A	0.9888	−0.1820	0.4556	0.034*	
H10B	1.0502	−0.2118	0.4289	0.034*	
C11A	0.97417 (11)	−0.1337 (3)	0.38977 (7)	0.0261 (5)	
H11A	0.9610	−0.2541	0.3830	0.031*	
H11B	1.0004	−0.0931	0.3664	0.031*	
C12A	0.91474 (10)	−0.0133 (3)	0.39151 (7)	0.0201 (4)	
C13A	0.88602 (10)	0.0015 (3)	0.34454 (7)	0.0177 (4)	
H13A	0.9214	0.0262	0.3256	0.021*	
C14A	0.85598 (11)	−0.1719 (3)	0.32792 (7)	0.0244 (5)	
H14A	0.8240	−0.2086	0.3480	0.029*	
H14B	0.8881	−0.2636	0.3278	0.029*	
C15A	0.82575 (11)	−0.1583 (3)	0.28219 (7)	0.0230 (5)	
H15A	0.8585	−0.1397	0.2612	0.028*	
H15B	0.8046	−0.2692	0.2751	0.028*	
C16A	0.77796 (10)	−0.0055 (3)	0.27877 (7)	0.0181 (4)	
C17A	0.75539 (10)	0.0474 (3)	0.23176 (7)	0.0186 (4)	
H17A	0.7920	0.0380	0.2131	0.022*	
C18A	0.70145 (10)	−0.0573 (3)	0.20946 (7)	0.0223 (5)	
H18A	0.6635	−0.0402	0.2265	0.027*	
C19A	0.68780 (10)	0.0178 (3)	0.16346 (7)	0.0244 (5)	
H19A	0.6894	0.1464	0.1649	0.029*	
H19B	0.7210	−0.0208	0.1445	0.029*	
C20A	0.62416 (11)	−0.0373 (3)	0.14305 (7)	0.0255 (5)	
H20A	0.6229	−0.1655	0.1401	0.031*	
H20B	0.5906	−0.0022	0.1621	0.031*	
C21A	0.61307 (11)	0.0479 (4)	0.09841 (7)	0.0314 (6)	
H21A	0.6432	−0.0013	0.0785	0.038*	
H21B	0.6214	0.1742	0.1008	0.038*	
C22A	0.54642 (12)	0.0221 (4)	0.07873 (8)	0.0357 (6)	
H22A	0.5163	0.0687	0.0995	0.043*	
C23A	0.53055 (14)	−0.1730 (5)	0.07063 (9)	0.0496 (6)	
H23A	0.4880	−0.1827	0.0595	0.074*	
H23B	0.5347	−0.2377	0.0974	0.074*	
H23C	0.5589	−0.2212	0.0499	0.074*	
C24A	0.53904 (13)	0.1299 (5)	0.03664 (8)	0.0496 (6)	
H24A	0.4965	0.1194	0.0255	0.074*	
H24B	0.5677	0.0855	0.0156	0.074*	
H24C	0.5483	0.2525	0.0425	0.074*	
C25A	0.71424 (11)	−0.2564 (3)	0.20705 (7)	0.0263 (5)	
H25A	0.7148	−0.3057	0.2358	0.039*	
H25B	0.7543	−0.2762	0.1941	0.039*	
H25C	0.6817	−0.3126	0.1897	0.039*	
C26A	0.72068 (10)	−0.0403 (3)	0.30730 (7)	0.0271 (5)	
H26A	0.7345	−0.0505	0.3371	0.041*	
H26B	0.7004	−0.1485	0.2982	0.041*	
H26C	0.6914	0.0566	0.3043	0.041*	
C27A	0.86742 (11)	−0.0928 (3)	0.42317 (7)	0.0288 (5)	
H27A	0.8283	−0.0293	0.4207	0.043*	
H27B	0.8841	−0.0833	0.4524	0.043*	
H27C	0.8604	−0.2156	0.4162	0.043*	
Cl1B	0.58765 (3)	0.96469 (11)	0.48748 (2)	0.04346 (18)	
C1B	0.22872 (11)	1.2289 (3)	0.24711 (7)	0.0242 (5)	
H1BA	0.2428	1.3033	0.2235	0.029*	
H1BB	0.1834	1.2425	0.2494	0.029*	
C2B	0.26239 (11)	1.2824 (3)	0.29016 (7)	0.0251 (5)	
H2BA	0.2871	1.3895	0.2866	0.030*	
H2BB	0.2322	1.3018	0.3129	0.030*	
C3B	0.30473 (10)	1.1239 (3)	0.30081 (7)	0.0192 (4)	
H3BA	0.3411	1.1346	0.2819	0.023*	
C4B	0.33183 (10)	1.1071 (3)	0.34692 (7)	0.0202 (4)	
H4BA	0.2973	1.0866	0.3669	0.024*	
C5B	0.36654 (11)	1.2778 (3)	0.36017 (7)	0.0249 (5)	
H5BA	0.3891	1.3223	0.3354	0.030*	
H5BB	0.3357	1.3667	0.3680	0.030*	
C6B	0.41218 (12)	1.2533 (3)	0.39736 (7)	0.0286 (5)	
H6BA	0.4288	1.3548	0.4105	0.034*	
C7B	0.43094 (11)	1.0964 (3)	0.41318 (7)	0.0256 (5)	
C8B	0.47725 (12)	1.0892 (4)	0.45172 (7)	0.0335 (6)	
H8BA	0.4928	1.2077	0.4582	0.040*	
H8BB	0.4560	1.0454	0.4771	0.040*	
C9B	0.53163 (12)	0.9694 (4)	0.44172 (7)	0.0327 (6)	
H9BA	0.5530	1.0167	0.4163	0.039*	
C10B	0.50845 (12)	0.7855 (3)	0.43114 (7)	0.0325 (6)	
H10C	0.4853	0.7383	0.4553	0.039*	
H10D	0.5440	0.7077	0.4260	0.039*	
C11B	0.46522 (11)	0.7936 (3)	0.39023 (7)	0.0278 (5)	
H11C	0.4500	0.6749	0.3837	0.033*	
H11D	0.4898	0.8329	0.3659	0.033*	
C12B	0.40789 (11)	0.9190 (3)	0.39480 (7)	0.0214 (5)	
C13B	0.37703 (10)	0.9468 (3)	0.34883 (6)	0.0199 (4)	
H13B	0.4115	0.9746	0.3293	0.024*	
C14B	0.34475 (11)	0.7802 (3)	0.33041 (7)	0.0245 (5)	
H14C	0.3129	0.7420	0.3505	0.029*	
H14D	0.3758	0.6862	0.3287	0.029*	
C15B	0.31333 (11)	0.8052 (3)	0.28510 (7)	0.0244 (5)	
H15C	0.3457	0.8254	0.2639	0.029*	
H15D	0.2910	0.6974	0.2769	0.029*	
C16B	0.26710 (10)	0.9615 (3)	0.28410 (6)	0.0183 (4)	
C17B	0.24560 (10)	1.0311 (3)	0.23834 (7)	0.0190 (4)	
H17B	0.2828	1.0306	0.2201	0.023*	
C18B	0.19237 (10)	0.9343 (3)	0.21285 (7)	0.0208 (4)	
H18B	0.1534	0.9479	0.2290	0.025*	
C19B	0.18222 (10)	1.0233 (3)	0.16803 (7)	0.0236 (5)	
H19C	0.1846	1.1510	0.1717	0.028*	
H19D	0.2163	0.9881	0.1494	0.028*	
C20B	0.11952 (11)	0.9775 (3)	0.14522 (7)	0.0254 (5)	
H20C	0.0854	1.0037	0.1646	0.030*	
H20D	0.1185	0.8512	0.1391	0.030*	
C21B	0.10853 (10)	1.0795 (3)	0.10277 (7)	0.0246 (5)	
H21C	0.1401	1.0427	0.0823	0.030*	
H21D	0.1148	1.2050	0.1085	0.030*	
C22B	0.04329 (11)	1.0542 (4)	0.08146 (7)	0.0281 (5)	
H22B	0.0118	1.0797	0.1034	0.034*	
C23B	0.03199 (12)	0.8655 (4)	0.06518 (8)	0.0362 (6)	
H23D	0.0382	0.7835	0.0888	0.054*	
H23E	0.0611	0.8388	0.0427	0.054*	
H23F	−0.0104	0.8552	0.0538	0.054*	
C24B	0.03354 (13)	1.1879 (4)	0.04411 (8)	0.0409 (7)	
H24D	−0.0074	1.1705	0.0308	0.061*	
H24E	0.0653	1.1701	0.0229	0.061*	
H24F	0.0366	1.3066	0.0554	0.061*	
C25B	0.20475 (11)	0.7357 (3)	0.20730 (8)	0.0280 (5)	
H25D	0.2050	0.6789	0.2352	0.042*	
H25E	0.2449	0.7188	0.1941	0.042*	
H25F	0.1721	0.6847	0.1891	0.042*	
C26B	0.20904 (10)	0.9216 (3)	0.31165 (7)	0.0256 (5)	
H26D	0.2224	0.9011	0.3413	0.038*	
H26E	0.1879	0.8179	0.3005	0.038*	
H26F	0.1807	1.0210	0.3104	0.038*	
C27B	0.36112 (11)	0.8355 (3)	0.42662 (7)	0.0311 (5)	
H27D	0.3792	0.8377	0.4555	0.047*	
H27E	0.3526	0.7150	0.4182	0.047*	
H27F	0.3225	0.9021	0.4259	0.047*	
Cl1C	0.18518 (3)	0.58349 (10)	0.03645 (2)	0.04151 (17)	
C1C	0.55598 (12)	0.6996 (3)	0.27704 (7)	0.0294 (5)	
H1CA	0.5456	0.7780	0.3008	0.035*	
H1CB	0.6015	0.6941	0.2750	0.035*	
C2C	0.52627 (12)	0.7685 (3)	0.23416 (7)	0.0289 (5)	
H2CA	0.5087	0.8864	0.2378	0.035*	
H2CB	0.5570	0.7718	0.2114	0.035*	
C3C	0.47454 (10)	0.6332 (3)	0.22383 (7)	0.0203 (5)	
H3CA	0.4403	0.6595	0.2436	0.024*	
C4C	0.44476 (10)	0.6342 (3)	0.17825 (7)	0.0204 (4)	
H4CA	0.4773	0.6059	0.1573	0.024*	
C5C	0.41697 (11)	0.8168 (3)	0.16725 (7)	0.0257 (5)	
H5CA	0.3976	0.8647	0.1930	0.031*	
H5CB	0.4510	0.8960	0.1595	0.031*	
C6C	0.36883 (11)	0.8136 (3)	0.13077 (7)	0.0273 (5)	
H6CA	0.3552	0.9222	0.1196	0.033*	
C7C	0.34414 (11)	0.6671 (3)	0.11313 (7)	0.0239 (5)	
C8C	0.29729 (12)	0.6809 (3)	0.07503 (7)	0.0305 (5)	
H8CA	0.2847	0.8038	0.0711	0.037*	
H8CB	0.3172	0.6417	0.0487	0.037*	
C9C	0.23974 (11)	0.5686 (4)	0.08278 (7)	0.0300 (5)	
H9CA	0.2187	0.6138	0.1085	0.036*	
C10C	0.25793 (11)	0.3783 (3)	0.09078 (7)	0.0266 (5)	
H10E	0.2796	0.3316	0.0658	0.032*	
H10F	0.2204	0.3073	0.0951	0.032*	
C11C	0.30124 (10)	0.3686 (3)	0.13120 (7)	0.0236 (5)	
H11E	0.3130	0.2456	0.1361	0.028*	
H11F	0.2777	0.4075	0.1561	0.028*	
C12C	0.36221 (10)	0.4814 (3)	0.12880 (7)	0.0199 (4)	
C13C	0.39273 (10)	0.4923 (3)	0.17505 (6)	0.0185 (4)	
H13C	0.3594	0.5305	0.1945	0.022*	
C14C	0.41661 (11)	0.3114 (3)	0.19231 (7)	0.0227 (5)	
H14E	0.3813	0.2299	0.1934	0.027*	
H14F	0.4465	0.2634	0.1721	0.027*	
C15C	0.44823 (10)	0.3217 (3)	0.23754 (7)	0.0216 (4)	
H15E	0.4171	0.3558	0.2586	0.026*	
H15F	0.4642	0.2053	0.2456	0.026*	
C16C	0.50254 (10)	0.4556 (3)	0.23895 (7)	0.0206 (4)	
C17C	0.52849 (10)	0.5104 (3)	0.28481 (7)	0.0212 (5)	
H17C	0.4920	0.5246	0.3034	0.025*	
C18C	0.57611 (11)	0.3911 (3)	0.30947 (7)	0.0245 (5)	
H18C	0.6153	0.3893	0.2933	0.029*	
C19C	0.59045 (11)	0.4728 (4)	0.35464 (7)	0.0309 (5)	
H19E	0.5914	0.6009	0.3515	0.037*	
H19F	0.5558	0.4441	0.3734	0.037*	
C20C	0.65131 (13)	0.4150 (5)	0.37731 (8)	0.0473 (8)	
H20E	0.6861	0.4356	0.3581	0.057*	
H20F	0.6493	0.2885	0.3830	0.057*	
C21C	0.66456 (12)	0.5128 (4)	0.42020 (8)	0.0398 (7)	
H21E	0.6571	0.6385	0.4154	0.048*	
H21F	0.6343	0.4725	0.4412	0.048*	
C22C	0.72962 (15)	0.4908 (5)	0.43994 (10)	0.0591 (10)	
H22C	0.7603	0.5287	0.4185	0.071*	
C23C	0.74359 (17)	0.2999 (6)	0.45201 (10)	0.0720 (8)	
H23G	0.7405	0.2270	0.4265	0.108*	
H23H	0.7137	0.2600	0.4728	0.108*	
H23I	0.7855	0.2917	0.4644	0.108*	
C24C	0.73709 (16)	0.6104 (6)	0.48006 (10)	0.0720 (8)	
H24G	0.7296	0.7317	0.4718	0.108*	
H24H	0.7791	0.5989	0.4921	0.108*	
H24I	0.7072	0.5756	0.5014	0.108*	
C25C	0.55354 (11)	0.1996 (3)	0.31474 (7)	0.0302 (5)	
H25G	0.5512	0.1430	0.2868	0.045*	
H25H	0.5126	0.1994	0.3273	0.045*	
H25I	0.5827	0.1362	0.3334	0.045*	
C26C	0.55701 (10)	0.3922 (3)	0.21070 (7)	0.0261 (5)	
H26G	0.5426	0.3845	0.1809	0.039*	
H26H	0.5711	0.2775	0.2205	0.039*	
H26I	0.5913	0.4752	0.2131	0.039*	
C27C	0.40669 (11)	0.3976 (3)	0.09571 (7)	0.0272 (5)	
H27G	0.4460	0.4608	0.0962	0.041*	
H27H	0.3876	0.4045	0.0671	0.041*	
H27I	0.4141	0.2755	0.1032	0.041*	
Cl1D	0.64629 (3)	0.57400 (10)	−0.01265 (2)	0.03807 (15)	
C1D	1.00060 (12)	0.7448 (3)	0.23690 (8)	0.0278 (5)	
H1DA	0.9858	0.8092	0.2620	0.033*	
H1DB	1.0459	0.7601	0.2354	0.033*	
C2D	0.96773 (11)	0.8136 (3)	0.19510 (8)	0.0274 (5)	
H2DA	0.9433	0.9194	0.2009	0.033*	
H2DB	0.9983	0.8408	0.1732	0.033*	
C3D	0.92505 (10)	0.6614 (3)	0.18048 (7)	0.0206 (4)	
H3DA	0.8886	0.6657	0.1994	0.025*	
C4D	0.89802 (10)	0.6608 (3)	0.13449 (7)	0.0210 (4)	
H4DA	0.9326	0.6447	0.1143	0.025*	
C5D	0.86469 (11)	0.8370 (3)	0.12407 (7)	0.0255 (5)	
H5DA	0.8418	0.8747	0.1493	0.031*	
H5DB	0.8963	0.9266	0.1184	0.031*	
C6D	0.82008 (11)	0.8268 (3)	0.08613 (7)	0.0283 (5)	
H6DA	0.8051	0.9334	0.0747	0.034*	
C7D	0.79980 (11)	0.6782 (3)	0.06722 (7)	0.0247 (5)	
C8D	0.75520 (12)	0.6848 (3)	0.02810 (7)	0.0308 (5)	
H8DA	0.7772	0.6463	0.0025	0.037*	
H8DB	0.7412	0.8061	0.0233	0.037*	
C9D	0.69853 (11)	0.5666 (3)	0.03474 (7)	0.0284 (5)	
H9DA	0.6756	0.6103	0.0598	0.034*	
C10D	0.71959 (12)	0.3780 (3)	0.04381 (7)	0.0295 (5)	
H10G	0.7429	0.3330	0.0194	0.035*	
H10H	0.6831	0.3025	0.0476	0.035*	
C11D	0.76123 (11)	0.3749 (3)	0.08487 (7)	0.0243 (5)	
H11G	0.7742	0.2534	0.0905	0.029*	
H11H	0.7364	0.4136	0.1091	0.029*	
C12D	0.82078 (10)	0.4925 (3)	0.08281 (7)	0.0200 (4)	
C13D	0.85156 (10)	0.5043 (3)	0.12901 (7)	0.0192 (4)	
H13D	0.8172	0.5269	0.1490	0.023*	
C14D	0.88307 (11)	0.3305 (3)	0.14417 (7)	0.0238 (5)	
H14G	0.8515	0.2373	0.1439	0.029*	
H14H	0.9148	0.2979	0.1235	0.029*	
C15D	0.91425 (11)	0.3390 (3)	0.18956 (7)	0.0230 (5)	
H15G	0.9358	0.2277	0.1956	0.028*	
H15H	0.8819	0.3539	0.2110	0.028*	
C16D	0.96183 (10)	0.4925 (3)	0.19380 (7)	0.0202 (4)	
C17D	0.98369 (10)	0.5429 (3)	0.24058 (7)	0.0211 (4)	
H17D	0.9467	0.5343	0.2589	0.025*	
C18D	1.03759 (11)	0.4378 (3)	0.26369 (7)	0.0232 (5)	
H18D	1.0763	0.4575	0.2476	0.028*	
C19D	1.04849 (11)	0.5095 (3)	0.30998 (7)	0.0283 (5)	
H19G	1.0471	0.6382	0.3090	0.034*	
H19H	1.0140	0.4702	0.3277	0.034*	
C20D	1.11032 (11)	0.4532 (3)	0.33198 (7)	0.0278 (5)	
H20G	1.1449	0.4840	0.3134	0.033*	
H20H	1.1104	0.3252	0.3356	0.033*	
C21D	1.12071 (11)	0.5409 (3)	0.37616 (7)	0.0312 (6)	
H21G	1.0879	0.5006	0.3952	0.037*	
H21H	1.1157	0.6682	0.3726	0.037*	
C22D	1.18468 (12)	0.5059 (4)	0.39857 (8)	0.0331 (6)	
H22D	1.2175	0.5333	0.3778	0.040*	
C23D	1.19301 (13)	0.3143 (4)	0.41256 (8)	0.0419 (7)	
H23J	1.2350	0.2974	0.4243	0.063*	
H23K	1.1863	0.2378	0.3879	0.063*	
H23L	1.1630	0.2863	0.4344	0.063*	
C24D	1.19426 (14)	0.6280 (5)	0.43772 (9)	0.0531 (8)	
H24J	1.2343	0.6030	0.4516	0.080*	
H24K	1.1612	0.6083	0.4579	0.080*	
H24L	1.1933	0.7494	0.4283	0.080*	
C25D	1.02495 (12)	0.2379 (3)	0.26461 (8)	0.0280 (5)	
H25J	1.0259	0.1915	0.2356	0.042*	
H25K	0.9842	0.2164	0.2765	0.042*	
H25L	1.0568	0.1805	0.2823	0.042*	
C26D	1.01907 (11)	0.4608 (3)	0.16543 (7)	0.0271 (5)	
H26J	1.0054	0.4510	0.1356	0.041*	
H26K	1.0398	0.3529	0.1743	0.041*	
H26L	1.0479	0.5585	0.1686	0.041*	
C27D	0.86633 (11)	0.4144 (3)	0.04977 (7)	0.0285 (5)	
H27J	0.9063	0.4739	0.0524	0.043*	
H27K	0.8491	0.4306	0.0209	0.043*	
H27L	0.8720	0.2900	0.0554	0.043*	

### Atomic displacement parameters (Å^2^)

**Table:**

	*U*^11^	*U*^22^	*U*^33^	*U*^12^	*U*^13^	*U*^23^
Cl1A	0.0308 (3)	0.0500 (4)	0.0303 (3)	−0.0058 (3)	−0.0088 (2)	−0.0029 (3)
C1A	0.0237 (12)	0.0193 (11)	0.0285 (11)	0.0037 (9)	−0.0021 (9)	0.0033 (9)
C2A	0.0325 (13)	0.0159 (11)	0.0306 (12)	0.0030 (9)	−0.0032 (10)	−0.0001 (9)
C3A	0.0198 (11)	0.0133 (10)	0.0229 (10)	0.0024 (8)	0.0009 (9)	0.0003 (8)
C4A	0.0239 (12)	0.0152 (10)	0.0200 (10)	0.0029 (9)	−0.0004 (9)	−0.0020 (8)
C5A	0.0298 (13)	0.0134 (10)	0.0298 (12)	0.0033 (9)	−0.0008 (10)	−0.0034 (9)
C6A	0.0353 (14)	0.0196 (11)	0.0272 (12)	−0.0017 (10)	−0.0001 (10)	−0.0061 (9)
C7A	0.0282 (12)	0.0213 (11)	0.0209 (10)	−0.0010 (10)	−0.0008 (9)	−0.0058 (9)
C8A	0.0365 (14)	0.0281 (13)	0.0255 (11)	−0.0009 (11)	−0.0052 (10)	−0.0034 (10)
C9A	0.0258 (13)	0.0417 (15)	0.0216 (11)	−0.0034 (11)	−0.0036 (9)	−0.0008 (10)
C10A	0.0248 (13)	0.0291 (13)	0.0296 (12)	0.0057 (10)	−0.0052 (10)	−0.0044 (10)
C11A	0.0272 (13)	0.0236 (12)	0.0271 (11)	0.0059 (9)	−0.0062 (10)	−0.0052 (9)
C12A	0.0218 (11)	0.0161 (11)	0.0223 (10)	0.0028 (8)	−0.0018 (9)	−0.0005 (8)
C13A	0.0196 (11)	0.0128 (10)	0.0205 (10)	−0.0003 (8)	−0.0012 (8)	−0.0022 (8)
C14A	0.0289 (13)	0.0128 (10)	0.0310 (12)	0.0018 (9)	−0.0071 (10)	0.0007 (9)
C15A	0.0272 (12)	0.0125 (10)	0.0292 (11)	0.0005 (9)	−0.0049 (10)	−0.0016 (9)
C16A	0.0183 (11)	0.0154 (10)	0.0206 (10)	0.0005 (8)	−0.0005 (8)	0.0001 (8)
C17A	0.0165 (10)	0.0177 (11)	0.0218 (10)	−0.0003 (8)	0.0029 (8)	0.0011 (8)
C18A	0.0205 (11)	0.0216 (11)	0.0248 (11)	−0.0020 (9)	0.0004 (9)	0.0023 (9)
C19A	0.0224 (12)	0.0263 (12)	0.0246 (11)	−0.0027 (9)	0.0002 (9)	0.0052 (9)
C20A	0.0269 (12)	0.0269 (12)	0.0228 (11)	−0.0032 (10)	−0.0009 (9)	0.0046 (9)
C21A	0.0272 (13)	0.0428 (15)	0.0241 (11)	−0.0053 (11)	0.0003 (10)	0.0089 (11)
C22A	0.0259 (13)	0.0554 (18)	0.0257 (12)	0.0000 (12)	0.0006 (10)	0.0079 (11)
C23A	0.0375 (12)	0.0749 (16)	0.0360 (10)	−0.0074 (11)	−0.0074 (9)	0.0110 (11)
C24A	0.0375 (12)	0.0749 (16)	0.0360 (10)	−0.0074 (11)	−0.0074 (9)	0.0110 (11)
C25A	0.0271 (13)	0.0208 (11)	0.0308 (12)	−0.0041 (10)	−0.0035 (10)	−0.0009 (9)
C26A	0.0253 (12)	0.0305 (13)	0.0255 (11)	−0.0042 (10)	0.0018 (9)	0.0042 (10)
C27A	0.0285 (13)	0.0289 (13)	0.0289 (12)	0.0000 (10)	−0.0012 (10)	0.0057 (10)
Cl1B	0.0387 (4)	0.0554 (5)	0.0354 (3)	0.0023 (3)	−0.0163 (3)	−0.0031 (3)
C1B	0.0301 (13)	0.0174 (11)	0.0250 (11)	0.0023 (9)	−0.0026 (10)	0.0026 (9)
C2B	0.0340 (13)	0.0156 (11)	0.0255 (11)	0.0025 (9)	−0.0041 (10)	0.0008 (8)
C3B	0.0196 (11)	0.0149 (10)	0.0230 (10)	0.0007 (8)	−0.0008 (9)	0.0009 (8)
C4B	0.0232 (11)	0.0167 (11)	0.0208 (10)	0.0021 (8)	−0.0011 (9)	0.0011 (8)
C5B	0.0339 (13)	0.0155 (11)	0.0251 (11)	−0.0005 (9)	−0.0041 (10)	−0.0010 (8)
C6B	0.0358 (14)	0.0226 (12)	0.0270 (12)	−0.0001 (10)	−0.0060 (10)	−0.0061 (9)
C7B	0.0311 (13)	0.0288 (12)	0.0168 (10)	0.0014 (10)	−0.0025 (9)	−0.0034 (9)
C8B	0.0407 (15)	0.0342 (14)	0.0251 (11)	0.0037 (12)	−0.0098 (10)	−0.0033 (11)
C9B	0.0313 (14)	0.0453 (15)	0.0211 (11)	0.0030 (12)	−0.0077 (10)	0.0012 (11)
C10B	0.0356 (14)	0.0365 (15)	0.0250 (12)	0.0117 (11)	−0.0084 (10)	−0.0020 (10)
C11B	0.0339 (14)	0.0275 (12)	0.0217 (11)	0.0089 (10)	−0.0064 (10)	−0.0028 (9)
C12B	0.0247 (12)	0.0211 (11)	0.0183 (10)	0.0039 (9)	−0.0003 (9)	0.0034 (8)
C13B	0.0232 (11)	0.0185 (11)	0.0180 (10)	0.0020 (9)	−0.0008 (9)	0.0023 (8)
C14B	0.0271 (12)	0.0174 (11)	0.0286 (11)	0.0019 (9)	−0.0070 (10)	0.0014 (9)
C15B	0.0308 (13)	0.0162 (11)	0.0259 (11)	0.0033 (9)	−0.0062 (10)	−0.0011 (9)
C16B	0.0199 (11)	0.0140 (10)	0.0208 (10)	0.0004 (8)	−0.0015 (8)	0.0021 (8)
C17B	0.0187 (11)	0.0179 (11)	0.0204 (10)	0.0003 (8)	−0.0002 (8)	0.0032 (8)
C18B	0.0199 (11)	0.0186 (11)	0.0237 (11)	−0.0017 (9)	−0.0019 (9)	0.0004 (9)
C19B	0.0227 (12)	0.0240 (12)	0.0238 (11)	−0.0028 (9)	−0.0036 (9)	0.0028 (9)
C20B	0.0240 (12)	0.0258 (12)	0.0260 (11)	−0.0040 (10)	−0.0038 (9)	0.0027 (9)
C21B	0.0217 (11)	0.0293 (12)	0.0228 (10)	0.0001 (10)	−0.0011 (9)	0.0010 (10)
C22B	0.0206 (11)	0.0451 (15)	0.0185 (10)	0.0056 (11)	0.0015 (9)	−0.0009 (10)
C23B	0.0285 (14)	0.0534 (17)	0.0266 (12)	−0.0073 (12)	−0.0029 (11)	−0.0065 (12)
C24B	0.0381 (16)	0.0549 (18)	0.0294 (13)	0.0145 (13)	−0.0041 (12)	0.0032 (12)
C25B	0.0310 (13)	0.0203 (11)	0.0324 (12)	−0.0014 (10)	−0.0059 (10)	−0.0025 (10)
C26B	0.0261 (12)	0.0267 (12)	0.0240 (11)	−0.0031 (9)	−0.0005 (9)	0.0051 (9)
C27B	0.0331 (14)	0.0358 (14)	0.0245 (11)	−0.0018 (11)	0.0005 (10)	0.0086 (10)
Cl1C	0.0342 (4)	0.0513 (4)	0.0384 (3)	0.0062 (3)	−0.0129 (3)	0.0088 (3)
C1C	0.0327 (14)	0.0298 (13)	0.0256 (12)	−0.0129 (10)	−0.0029 (10)	−0.0048 (10)
C2C	0.0359 (14)	0.0236 (12)	0.0272 (12)	−0.0128 (10)	−0.0009 (10)	−0.0009 (9)
C3C	0.0243 (12)	0.0146 (10)	0.0222 (10)	−0.0053 (8)	0.0025 (9)	−0.0021 (8)
C4C	0.0253 (12)	0.0154 (10)	0.0206 (10)	−0.0035 (9)	0.0016 (9)	−0.0003 (8)
C5C	0.0367 (14)	0.0160 (11)	0.0243 (11)	−0.0024 (10)	0.0008 (10)	0.0019 (9)
C6C	0.0351 (14)	0.0185 (11)	0.0280 (12)	0.0009 (10)	−0.0022 (10)	0.0055 (9)
C7C	0.0238 (12)	0.0242 (12)	0.0236 (11)	0.0024 (10)	0.0006 (9)	0.0055 (9)
C8C	0.0356 (14)	0.0284 (13)	0.0272 (12)	0.0010 (11)	−0.0078 (10)	0.0070 (10)
C9C	0.0281 (13)	0.0395 (14)	0.0218 (11)	0.0050 (12)	−0.0071 (10)	0.0045 (11)
C10C	0.0217 (12)	0.0317 (13)	0.0262 (11)	−0.0030 (10)	−0.0055 (9)	0.0026 (10)
C11C	0.0226 (12)	0.0208 (11)	0.0270 (11)	−0.0037 (9)	−0.0056 (9)	0.0048 (9)
C12C	0.0208 (11)	0.0180 (11)	0.0209 (10)	−0.0012 (8)	−0.0002 (8)	0.0012 (8)
C13C	0.0206 (11)	0.0146 (10)	0.0201 (10)	−0.0018 (8)	0.0001 (8)	−0.0004 (8)
C14C	0.0231 (12)	0.0136 (10)	0.0309 (12)	−0.0043 (9)	−0.0086 (9)	0.0004 (9)
C15C	0.0221 (11)	0.0154 (10)	0.0271 (11)	−0.0021 (9)	−0.0056 (9)	0.0030 (9)
C16C	0.0203 (11)	0.0205 (11)	0.0210 (10)	−0.0035 (9)	0.0010 (9)	−0.0034 (9)
C17C	0.0192 (11)	0.0258 (12)	0.0185 (10)	−0.0041 (9)	−0.0008 (8)	−0.0044 (8)
C18C	0.0177 (11)	0.0330 (13)	0.0225 (11)	0.0007 (9)	−0.0035 (9)	−0.0069 (9)
C19C	0.0263 (13)	0.0426 (15)	0.0233 (11)	0.0027 (11)	−0.0066 (10)	−0.0076 (11)
C20C	0.0293 (15)	0.080 (2)	0.0320 (14)	0.0174 (15)	−0.0116 (11)	−0.0175 (14)
C21C	0.0305 (14)	0.0605 (19)	0.0280 (13)	0.0072 (13)	−0.0070 (11)	−0.0030 (12)
C22C	0.0389 (17)	0.099 (3)	0.0383 (16)	0.0113 (18)	−0.0119 (13)	−0.0124 (17)
C23C	0.0594 (16)	0.110 (2)	0.0457 (13)	0.0200 (16)	−0.0185 (12)	−0.0137 (14)
C24C	0.0594 (16)	0.110 (2)	0.0457 (13)	0.0200 (16)	−0.0185 (12)	−0.0137 (14)
C25C	0.0282 (13)	0.0340 (14)	0.0282 (12)	0.0060 (10)	−0.0062 (10)	−0.0005 (10)
C26C	0.0213 (12)	0.0304 (13)	0.0266 (11)	0.0011 (9)	−0.0006 (9)	−0.0082 (9)
C27C	0.0255 (12)	0.0289 (13)	0.0272 (11)	0.0012 (10)	−0.0008 (10)	−0.0053 (10)
Cl1D	0.0311 (3)	0.0467 (4)	0.0359 (3)	0.0050 (3)	−0.0079 (3)	0.0077 (3)
C1D	0.0278 (13)	0.0167 (11)	0.0388 (13)	−0.0016 (9)	−0.0034 (11)	−0.0062 (10)
C2D	0.0289 (13)	0.0147 (11)	0.0384 (13)	−0.0046 (9)	−0.0011 (10)	−0.0012 (9)
C3D	0.0221 (11)	0.0113 (10)	0.0284 (11)	0.0003 (9)	0.0028 (9)	0.0000 (8)
C4D	0.0225 (11)	0.0128 (10)	0.0277 (11)	0.0001 (9)	0.0033 (9)	0.0011 (8)
C5D	0.0310 (13)	0.0118 (10)	0.0342 (12)	0.0004 (9)	0.0064 (10)	0.0028 (9)
C6D	0.0325 (14)	0.0204 (11)	0.0320 (12)	0.0043 (10)	−0.0001 (10)	0.0056 (10)
C7D	0.0284 (12)	0.0204 (11)	0.0256 (11)	0.0014 (9)	0.0032 (10)	0.0050 (9)
C8D	0.0382 (15)	0.0233 (12)	0.0306 (12)	0.0029 (10)	−0.0022 (11)	0.0057 (10)
C9D	0.0244 (12)	0.0352 (14)	0.0255 (11)	0.0062 (11)	−0.0017 (9)	0.0047 (11)
C10D	0.0293 (13)	0.0284 (13)	0.0307 (12)	−0.0041 (10)	0.0001 (10)	0.0038 (10)
C11D	0.0266 (12)	0.0211 (11)	0.0254 (11)	−0.0018 (9)	0.0013 (10)	0.0055 (9)
C12D	0.0226 (11)	0.0159 (10)	0.0215 (10)	−0.0005 (8)	0.0013 (9)	0.0024 (8)
C13D	0.0227 (11)	0.0113 (10)	0.0237 (11)	0.0008 (8)	0.0017 (9)	0.0028 (8)
C14D	0.0288 (13)	0.0109 (10)	0.0316 (12)	0.0006 (9)	−0.0024 (10)	−0.0007 (9)
C15D	0.0287 (13)	0.0102 (10)	0.0300 (11)	0.0005 (9)	−0.0021 (10)	0.0024 (9)
C16D	0.0220 (11)	0.0138 (10)	0.0247 (11)	0.0025 (8)	−0.0010 (9)	−0.0001 (8)
C17D	0.0216 (11)	0.0150 (11)	0.0268 (11)	−0.0001 (9)	0.0001 (9)	−0.0030 (8)
C18D	0.0229 (12)	0.0221 (11)	0.0245 (11)	0.0020 (9)	−0.0009 (9)	−0.0007 (9)
C19D	0.0264 (13)	0.0275 (13)	0.0309 (12)	0.0023 (10)	0.0022 (10)	−0.0044 (10)
C20D	0.0259 (12)	0.0281 (12)	0.0294 (12)	0.0019 (10)	−0.0016 (10)	−0.0057 (10)
C21D	0.0272 (13)	0.0364 (14)	0.0298 (12)	−0.0034 (11)	−0.0013 (10)	−0.0068 (10)
C22D	0.0299 (14)	0.0417 (15)	0.0275 (12)	−0.0023 (11)	−0.0009 (10)	−0.0050 (11)
C23D	0.0378 (16)	0.0595 (19)	0.0282 (13)	−0.0004 (14)	−0.0033 (12)	0.0087 (13)
C24D	0.0451 (18)	0.072 (2)	0.0421 (16)	−0.0048 (16)	−0.0098 (14)	−0.0170 (16)
C25D	0.0344 (14)	0.0190 (11)	0.0304 (12)	0.0024 (10)	−0.0039 (10)	0.0006 (9)
C26D	0.0267 (12)	0.0247 (12)	0.0298 (12)	0.0039 (10)	0.0028 (10)	−0.0015 (10)
C27D	0.0283 (13)	0.0305 (13)	0.0268 (11)	0.0023 (10)	0.0021 (10)	−0.0023 (10)

### Geometric parameters (Å, °)

**Table:**

Cl1A—C9A	1.817 (2)	Cl1C—C9C	1.817 (2)
C1A—C2A	1.545 (3)	C1C—C2C	1.538 (3)
C1A—C17A	1.560 (3)	C1C—C17C	1.560 (3)
C1A—H1AA	0.9700	C1C—H1CA	0.9700
C1A—H1AB	0.9700	C1C—H1CB	0.9700
C2A—C3A	1.526 (3)	C2C—C3C	1.525 (3)
C2A—H2AA	0.9700	C2C—H2CA	0.9700
C2A—H2AB	0.9700	C2C—H2CB	0.9700
C3A—C4A	1.525 (3)	C3C—C4C	1.524 (3)
C3A—C16A	1.534 (3)	C3C—C16C	1.531 (3)
C3A—H3AA	0.9800	C3C—H3CA	0.9800
C4A—C5A	1.530 (3)	C4C—C5C	1.531 (3)
C4A—C13A	1.545 (3)	C4C—C13C	1.537 (3)
C4A—H4AA	0.9800	C4C—H4CA	0.9800
C5A—C6A	1.489 (3)	C5C—C6C	1.499 (3)
C5A—H5AA	0.9700	C5C—H5CA	0.9700
C5A—H5AB	0.9700	C5C—H5CB	0.9700
C6A—C7A	1.339 (3)	C6C—C7C	1.331 (3)
C6A—H6AA	0.9300	C6C—H6CA	0.9300
C7A—C8A	1.518 (3)	C7C—C8C	1.521 (3)
C7A—C12A	1.519 (3)	C7C—C12C	1.525 (3)
C8A—C9A	1.517 (3)	C8C—C9C	1.509 (3)
C8A—H8AA	0.9700	C8C—H8CA	0.9700
C8A—H8AB	0.9700	C8C—H8CB	0.9700
C9A—C10A	1.510 (3)	C9C—C10C	1.502 (3)
C9A—H9AA	0.9800	C9C—H9CA	0.9800
C10A—C11A	1.531 (3)	C10C—C11C	1.529 (3)
C10A—H10A	0.9700	C10C—H10E	0.9700
C10A—H10B	0.9700	C10C—H10F	0.9700
C11A—C12A	1.555 (3)	C11C—C12C	1.550 (3)
C11A—H11A	0.9700	C11C—H11E	0.9700
C11A—H11B	0.9700	C11C—H11F	0.9700
C12A—C27A	1.538 (3)	C12C—C27C	1.541 (3)
C12A—C13A	1.558 (3)	C12C—C13C	1.551 (3)
C13A—C14A	1.535 (3)	C13C—C14C	1.543 (3)
C13A—H13A	0.9800	C13C—H13C	0.9800
C14A—C15A	1.535 (3)	C14C—C15C	1.532 (3)
C14A—H14A	0.9700	C14C—H14E	0.9700
C14A—H14B	0.9700	C14C—H14F	0.9700
C15A—C16A	1.535 (3)	C15C—C16C	1.531 (3)
C15A—H15A	0.9700	C15C—H15E	0.9700
C15A—H15B	0.9700	C15C—H15F	0.9700
C16A—C26A	1.539 (3)	C16C—C26C	1.539 (3)
C16A—C17A	1.564 (3)	C16C—C17C	1.558 (3)
C17A—C18A	1.537 (3)	C17C—C18C	1.537 (3)
C17A—H17A	0.9800	C17C—H17C	0.9800
C18A—C25A	1.526 (3)	C18C—C25C	1.529 (3)
C18A—C19A	1.545 (3)	C18C—C19C	1.543 (3)
C18A—H18A	0.9800	C18C—H18C	0.9800
C19A—C20A	1.531 (3)	C19C—C20C	1.515 (3)
C19A—H19A	0.9700	C19C—H19E	0.9700
C19A—H19B	0.9700	C19C—H19F	0.9700
C20A—C21A	1.529 (3)	C20C—C21C	1.531 (4)
C20A—H20A	0.9700	C20C—H20E	0.9700
C20A—H20B	0.9700	C20C—H20F	0.9700
C21A—C22A	1.536 (3)	C21C—C22C	1.503 (4)
C21A—H21A	0.9700	C21C—H21E	0.9700
C21A—H21B	0.9700	C21C—H21F	0.9700
C22A—C23A	1.526 (4)	C22C—C23C	1.512 (5)
C22A—C24A	1.534 (4)	C22C—C24C	1.533 (5)
C22A—H22A	0.9800	C22C—H22C	0.9800
C23A—H23A	0.9600	C23C—H23G	0.9600
C23A—H23B	0.9600	C23C—H23H	0.9600
C23A—H23C	0.9600	C23C—H23I	0.9600
C24A—H24A	0.9600	C24C—H24G	0.9600
C24A—H24B	0.9600	C24C—H24H	0.9600
C24A—H24C	0.9600	C24C—H24I	0.9600
C25A—H25A	0.9600	C25C—H25G	0.9600
C25A—H25B	0.9600	C25C—H25H	0.9600
C25A—H25C	0.9600	C25C—H25I	0.9600
C26A—H26A	0.9600	C26C—H26G	0.9600
C26A—H26B	0.9600	C26C—H26H	0.9600
C26A—H26C	0.9600	C26C—H26I	0.9600
C27A—H27A	0.9600	C27C—H27G	0.9600
C27A—H27B	0.9600	C27C—H27H	0.9600
C27A—H27C	0.9600	C27C—H27I	0.9600
Cl1B—C9B	1.820 (2)	Cl1D—C9D	1.810 (2)
C1B—C2B	1.543 (3)	C1D—C2D	1.537 (3)
C1B—C17B	1.557 (3)	C1D—C17D	1.566 (3)
C1B—H1BA	0.9700	C1D—H1DA	0.9700
C1B—H1BB	0.9700	C1D—H1DB	0.9700
C2B—C3B	1.524 (3)	C2D—C3D	1.521 (3)
C2B—H2BA	0.9700	C2D—H2DA	0.9700
C2B—H2BB	0.9700	C2D—H2DB	0.9700
C3B—C4B	1.523 (3)	C3D—C4D	1.514 (3)
C3B—C16B	1.541 (3)	C3D—C16D	1.542 (3)
C3B—H3BA	0.9800	C3D—H3DA	0.9800
C4B—C5B	1.531 (3)	C4D—C5D	1.534 (3)
C4B—C13B	1.542 (3)	C4D—C13D	1.542 (3)
C4B—H4BA	0.9800	C4D—H4DA	0.9800
C5B—C6B	1.492 (3)	C5D—C6D	1.486 (3)
C5B—H5BA	0.9700	C5D—H5DA	0.9700
C5B—H5BB	0.9700	C5D—H5DB	0.9700
C6B—C7B	1.335 (3)	C6D—C7D	1.327 (3)
C6B—H6BA	0.9300	C6D—H6DA	0.9300
C7B—C8B	1.522 (3)	C7D—C8D	1.513 (3)
C7B—C12B	1.526 (3)	C7D—C12D	1.541 (3)
C8B—C9B	1.502 (3)	C8D—C9D	1.514 (3)
C8B—H8BA	0.9700	C8D—H8DA	0.9700
C8B—H8BB	0.9700	C8D—H8DB	0.9700
C9B—C10B	1.503 (4)	C9D—C10D	1.513 (3)
C9B—H9BA	0.9800	C9D—H9DA	0.9800
C10B—C11B	1.540 (3)	C10D—C11D	1.523 (3)
C10B—H10C	0.9700	C10D—H10G	0.9700
C10B—H10D	0.9700	C10D—H10H	0.9700
C11B—C12B	1.549 (3)	C11D—C12D	1.545 (3)
C11B—H11C	0.9700	C11D—H11G	0.9700
C11B—H11D	0.9700	C11D—H11H	0.9700
C12B—C27B	1.545 (3)	C12D—C27D	1.537 (3)
C12B—C13B	1.558 (3)	C12D—C13D	1.552 (3)
C13B—C14B	1.531 (3)	C13D—C14D	1.537 (3)
C13B—H13B	0.9800	C13D—H13D	0.9800
C14B—C15B	1.542 (3)	C14D—C15D	1.532 (3)
C14B—H14C	0.9700	C14D—H14G	0.9700
C14B—H14D	0.9700	C14D—H14H	0.9700
C15B—C16B	1.532 (3)	C15D—C16D	1.538 (3)
C15B—H15C	0.9700	C15D—H15G	0.9700
C15B—H15D	0.9700	C15D—H15H	0.9700
C16B—C26B	1.542 (3)	C16D—C26D	1.532 (3)
C16B—C17B	1.560 (3)	C16D—C17D	1.548 (3)
C17B—C18B	1.542 (3)	C17D—C18D	1.549 (3)
C17B—H17B	0.9800	C17D—H17D	0.9800
C18B—C25B	1.529 (3)	C18D—C25D	1.529 (3)
C18B—C19B	1.544 (3)	C18D—C19D	1.536 (3)
C18B—H18B	0.9800	C18D—H18D	0.9800
C19B—C20B	1.527 (3)	C19D—C20D	1.521 (3)
C19B—H19C	0.9700	C19D—H19G	0.9700
C19B—H19D	0.9700	C19D—H19H	0.9700
C20B—C21B	1.528 (3)	C20D—C21D	1.523 (3)
C20B—H20C	0.9700	C20D—H20G	0.9700
C20B—H20D	0.9700	C20D—H20H	0.9700
C21B—C22B	1.528 (3)	C21D—C22D	1.529 (3)
C21B—H21C	0.9700	C21D—H21G	0.9700
C21B—H21D	0.9700	C21D—H21H	0.9700
C22B—C23B	1.524 (4)	C22D—C23D	1.515 (4)
C22B—C24B	1.538 (3)	C22D—C24D	1.525 (4)
C22B—H22B	0.9800	C22D—H22D	0.9800
C23B—H23D	0.9600	C23D—H23J	0.9600
C23B—H23E	0.9600	C23D—H23K	0.9600
C23B—H23F	0.9600	C23D—H23L	0.9600
C24B—H24D	0.9600	C24D—H24J	0.9600
C24B—H24E	0.9600	C24D—H24K	0.9600
C24B—H24F	0.9600	C24D—H24L	0.9600
C25B—H25D	0.9600	C25D—H25J	0.9600
C25B—H25E	0.9600	C25D—H25K	0.9600
C25B—H25F	0.9600	C25D—H25L	0.9600
C26B—H26D	0.9600	C26D—H26J	0.9600
C26B—H26E	0.9600	C26D—H26K	0.9600
C26B—H26F	0.9600	C26D—H26L	0.9600
C27B—H27D	0.9600	C27D—H27J	0.9600
C27B—H27E	0.9600	C27D—H27K	0.9600
C27B—H27F	0.9600	C27D—H27L	0.9600
			
C2A—C1A—C17A	107.46 (17)	C2C—C1C—C17C	107.05 (18)
C2A—C1A—H1AA	110.2	C2C—C1C—H1CA	110.3
C17A—C1A—H1AA	110.2	C17C—C1C—H1CA	110.3
C2A—C1A—H1AB	110.2	C2C—C1C—H1CB	110.3
C17A—C1A—H1AB	110.2	C17C—C1C—H1CB	110.3
H1AA—C1A—H1AB	108.5	H1CA—C1C—H1CB	108.6
C3A—C2A—C1A	103.55 (17)	C3C—C2C—C1C	103.32 (18)
C3A—C2A—H2AA	111.1	C3C—C2C—H2CA	111.1
C1A—C2A—H2AA	111.1	C1C—C2C—H2CA	111.1
C3A—C2A—H2AB	111.1	C3C—C2C—H2CB	111.1
C1A—C2A—H2AB	111.1	C1C—C2C—H2CB	111.1
H2AA—C2A—H2AB	109.0	H2CA—C2C—H2CB	109.1
C4A—C3A—C2A	118.48 (18)	C4C—C3C—C2C	117.89 (18)
C4A—C3A—C16A	114.66 (17)	C4C—C3C—C16C	115.64 (17)
C2A—C3A—C16A	105.04 (17)	C2C—C3C—C16C	104.41 (18)
C4A—C3A—H3AA	105.9	C4C—C3C—H3CA	106.0
C2A—C3A—H3AA	105.9	C2C—C3C—H3CA	106.0
C16A—C3A—H3AA	105.9	C16C—C3C—H3CA	106.0
C3A—C4A—C5A	110.65 (17)	C3C—C4C—C5C	110.87 (17)
C3A—C4A—C13A	108.85 (16)	C3C—C4C—C13C	109.62 (16)
C5A—C4A—C13A	110.50 (18)	C5C—C4C—C13C	109.78 (18)
C3A—C4A—H4AA	108.9	C3C—C4C—H4CA	108.8
C5A—C4A—H4AA	108.9	C5C—C4C—H4CA	108.8
C13A—C4A—H4AA	108.9	C13C—C4C—H4CA	108.8
C6A—C5A—C4A	113.12 (18)	C6C—C5C—C4C	113.74 (18)
C6A—C5A—H5AA	109.0	C6C—C5C—H5CA	108.8
C4A—C5A—H5AA	109.0	C4C—C5C—H5CA	108.8
C6A—C5A—H5AB	109.0	C6C—C5C—H5CB	108.8
C4A—C5A—H5AB	109.0	C4C—C5C—H5CB	108.8
H5AA—C5A—H5AB	107.8	H5CA—C5C—H5CB	107.7
C7A—C6A—C5A	125.0 (2)	C7C—C6C—C5C	124.9 (2)
C7A—C6A—H6AA	117.5	C7C—C6C—H6CA	117.5
C5A—C6A—H6AA	117.5	C5C—C6C—H6CA	117.5
C6A—C7A—C8A	120.0 (2)	C6C—C7C—C8C	120.0 (2)
C6A—C7A—C12A	123.30 (19)	C6C—C7C—C12C	122.5 (2)
C8A—C7A—C12A	116.69 (19)	C8C—C7C—C12C	117.40 (19)
C9A—C8A—C7A	110.71 (18)	C9C—C8C—C7C	110.81 (18)
C9A—C8A—H8AA	109.5	C9C—C8C—H8CA	109.5
C7A—C8A—H8AA	109.5	C7C—C8C—H8CA	109.5
C9A—C8A—H8AB	109.5	C9C—C8C—H8CB	109.5
C7A—C8A—H8AB	109.5	C7C—C8C—H8CB	109.5
H8AA—C8A—H8AB	108.1	H8CA—C8C—H8CB	108.1
C10A—C9A—C8A	110.8 (2)	C10C—C9C—C8C	110.8 (2)
C10A—C9A—Cl1A	110.15 (17)	C10C—C9C—Cl1C	110.09 (17)
C8A—C9A—Cl1A	109.71 (16)	C8C—C9C—Cl1C	110.06 (16)
C10A—C9A—H9AA	108.7	C10C—C9C—H9CA	108.6
C8A—C9A—H9AA	108.7	C8C—C9C—H9CA	108.6
Cl1A—C9A—H9AA	108.7	Cl1C—C9C—H9CA	108.6
C9A—C10A—C11A	108.83 (19)	C9C—C10C—C11C	108.98 (19)
C9A—C10A—H10A	109.9	C9C—C10C—H10E	109.9
C11A—C10A—H10A	109.9	C11C—C10C—H10E	109.9
C9A—C10A—H10B	109.9	C9C—C10C—H10F	109.9
C11A—C10A—H10B	109.9	C11C—C10C—H10F	109.9
H10A—C10A—H10B	108.3	H10E—C10C—H10F	108.3
C10A—C11A—C12A	113.79 (18)	C10C—C11C—C12C	114.81 (17)
C10A—C11A—H11A	108.8	C10C—C11C—H11E	108.6
C12A—C11A—H11A	108.8	C12C—C11C—H11E	108.6
C10A—C11A—H11B	108.8	C10C—C11C—H11F	108.6
C12A—C11A—H11B	108.8	C12C—C11C—H11F	108.6
H11A—C11A—H11B	107.7	H11E—C11C—H11F	107.5
C7A—C12A—C27A	109.15 (18)	C7C—C12C—C27C	108.63 (17)
C7A—C12A—C11A	108.05 (18)	C7C—C12C—C11C	108.31 (18)
C27A—C12A—C11A	109.72 (18)	C27C—C12C—C11C	109.43 (18)
C7A—C12A—C13A	110.21 (17)	C7C—C12C—C13C	109.72 (17)
C27A—C12A—C13A	111.70 (18)	C27C—C12C—C13C	112.34 (18)
C11A—C12A—C13A	107.94 (16)	C11C—C12C—C13C	108.33 (16)
C14A—C13A—C4A	111.51 (17)	C4C—C13C—C14C	111.27 (17)
C14A—C13A—C12A	113.41 (17)	C4C—C13C—C12C	112.20 (16)
C4A—C13A—C12A	112.57 (16)	C14C—C13C—C12C	113.28 (17)
C14A—C13A—H13A	106.2	C4C—C13C—H13C	106.5
C4A—C13A—H13A	106.2	C14C—C13C—H13C	106.5
C12A—C13A—H13A	106.2	C12C—C13C—H13C	106.5
C13A—C14A—C15A	114.12 (18)	C15C—C14C—C13C	113.63 (17)
C13A—C14A—H14A	108.7	C15C—C14C—H14E	108.8
C15A—C14A—H14A	108.7	C13C—C14C—H14E	108.8
C13A—C14A—H14B	108.7	C15C—C14C—H14F	108.8
C15A—C14A—H14B	108.7	C13C—C14C—H14F	108.8
H14A—C14A—H14B	107.6	H14E—C14C—H14F	107.7
C16A—C15A—C14A	111.89 (17)	C16C—C15C—C14C	111.76 (17)
C16A—C15A—H15A	109.2	C16C—C15C—H15E	109.3
C14A—C15A—H15A	109.2	C14C—C15C—H15E	109.3
C16A—C15A—H15B	109.2	C16C—C15C—H15F	109.3
C14A—C15A—H15B	109.2	C14C—C15C—H15F	109.3
H15A—C15A—H15B	107.9	H15E—C15C—H15F	107.9
C3A—C16A—C15A	106.01 (17)	C3C—C16C—C15C	106.34 (17)
C3A—C16A—C26A	112.15 (17)	C3C—C16C—C26C	112.96 (18)
C15A—C16A—C26A	111.26 (18)	C15C—C16C—C26C	110.75 (18)
C3A—C16A—C17A	100.70 (16)	C3C—C16C—C17C	99.78 (17)
C15A—C16A—C17A	116.12 (17)	C15C—C16C—C17C	116.64 (17)
C26A—C16A—C17A	110.10 (17)	C26C—C16C—C17C	109.93 (18)
C18A—C17A—C1A	112.08 (18)	C18C—C17C—C16C	120.35 (18)
C18A—C17A—C16A	119.31 (17)	C18C—C17C—C1C	111.47 (18)
C1A—C17A—C16A	103.47 (16)	C16C—C17C—C1C	103.15 (17)
C18A—C17A—H17A	107.1	C18C—C17C—H17C	107.0
C1A—C17A—H17A	107.1	C16C—C17C—H17C	107.0
C16A—C17A—H17A	107.1	C1C—C17C—H17C	107.0
C25A—C18A—C17A	113.24 (18)	C25C—C18C—C17C	113.57 (18)
C25A—C18A—C19A	110.17 (18)	C25C—C18C—C19C	109.6 (2)
C17A—C18A—C19A	109.96 (17)	C17C—C18C—C19C	108.88 (19)
C25A—C18A—H18A	107.8	C25C—C18C—H18C	108.2
C17A—C18A—H18A	107.8	C17C—C18C—H18C	108.2
C19A—C18A—H18A	107.8	C19C—C18C—H18C	108.2
C20A—C19A—C18A	114.78 (18)	C20C—C19C—C18C	116.5 (2)
C20A—C19A—H19A	108.6	C20C—C19C—H19E	108.2
C18A—C19A—H19A	108.6	C18C—C19C—H19E	108.2
C20A—C19A—H19B	108.6	C20C—C19C—H19F	108.2
C18A—C19A—H19B	108.6	C18C—C19C—H19F	108.2
H19A—C19A—H19B	107.5	H19E—C19C—H19F	107.3
C21A—C20A—C19A	111.64 (18)	C19C—C20C—C21C	113.3 (2)
C21A—C20A—H20A	109.3	C19C—C20C—H20E	108.9
C19A—C20A—H20A	109.3	C21C—C20C—H20E	108.9
C21A—C20A—H20B	109.3	C19C—C20C—H20F	108.9
C19A—C20A—H20B	109.3	C21C—C20C—H20F	108.9
H20A—C20A—H20B	108.0	H20E—C20C—H20F	107.7
C20A—C21A—C22A	114.87 (19)	C22C—C21C—C20C	116.2 (2)
C20A—C21A—H21A	108.6	C22C—C21C—H21E	108.2
C22A—C21A—H21A	108.6	C20C—C21C—H21E	108.2
C20A—C21A—H21B	108.6	C22C—C21C—H21F	108.2
C22A—C21A—H21B	108.6	C20C—C21C—H21F	108.2
H21A—C21A—H21B	107.5	H21E—C21C—H21F	107.4
C23A—C22A—C24A	110.8 (2)	C21C—C22C—C23C	112.1 (3)
C23A—C22A—C21A	112.5 (2)	C21C—C22C—C24C	109.6 (3)
C24A—C22A—C21A	109.8 (2)	C23C—C22C—C24C	110.2 (3)
C23A—C22A—H22A	107.9	C21C—C22C—H22C	108.3
C24A—C22A—H22A	107.9	C23C—C22C—H22C	108.3
C21A—C22A—H22A	107.9	C24C—C22C—H22C	108.3
C22A—C23A—H23A	109.5	C22C—C23C—H23G	109.5
C22A—C23A—H23B	109.5	C22C—C23C—H23H	109.5
H23A—C23A—H23B	109.5	H23G—C23C—H23H	109.5
C22A—C23A—H23C	109.5	C22C—C23C—H23I	109.5
H23A—C23A—H23C	109.5	H23G—C23C—H23I	109.5
H23B—C23A—H23C	109.5	H23H—C23C—H23I	109.5
C22A—C24A—H24A	109.5	C22C—C24C—H24G	109.5
C22A—C24A—H24B	109.5	C22C—C24C—H24H	109.5
H24A—C24A—H24B	109.5	H24G—C24C—H24H	109.5
C22A—C24A—H24C	109.5	C22C—C24C—H24I	109.5
H24A—C24A—H24C	109.5	H24G—C24C—H24I	109.5
H24B—C24A—H24C	109.5	H24H—C24C—H24I	109.5
C18A—C25A—H25A	109.5	C18C—C25C—H25G	109.5
C18A—C25A—H25B	109.5	C18C—C25C—H25H	109.5
H25A—C25A—H25B	109.5	H25G—C25C—H25H	109.5
C18A—C25A—H25C	109.5	C18C—C25C—H25I	109.5
H25A—C25A—H25C	109.5	H25G—C25C—H25I	109.5
H25B—C25A—H25C	109.5	H25H—C25C—H25I	109.5
C16A—C26A—H26A	109.5	C16C—C26C—H26G	109.5
C16A—C26A—H26B	109.5	C16C—C26C—H26H	109.5
H26A—C26A—H26B	109.5	H26G—C26C—H26H	109.5
C16A—C26A—H26C	109.5	C16C—C26C—H26I	109.5
H26A—C26A—H26C	109.5	H26G—C26C—H26I	109.5
H26B—C26A—H26C	109.5	H26H—C26C—H26I	109.5
C12A—C27A—H27A	109.5	C12C—C27C—H27G	109.5
C12A—C27A—H27B	109.5	C12C—C27C—H27H	109.5
H27A—C27A—H27B	109.5	H27G—C27C—H27H	109.5
C12A—C27A—H27C	109.5	C12C—C27C—H27I	109.5
H27A—C27A—H27C	109.5	H27G—C27C—H27I	109.5
H27B—C27A—H27C	109.5	H27H—C27C—H27I	109.5
C2B—C1B—C17B	107.18 (17)	C2D—C1D—C17D	106.72 (18)
C2B—C1B—H1BA	110.3	C2D—C1D—H1DA	110.4
C17B—C1B—H1BA	110.3	C17D—C1D—H1DA	110.4
C2B—C1B—H1BB	110.3	C2D—C1D—H1DB	110.4
C17B—C1B—H1BB	110.3	C17D—C1D—H1DB	110.4
H1BA—C1B—H1BB	108.5	H1DA—C1D—H1DB	108.6
C3B—C2B—C1B	103.74 (17)	C3D—C2D—C1D	104.33 (18)
C3B—C2B—H2BA	111.0	C3D—C2D—H2DA	110.9
C1B—C2B—H2BA	111.0	C1D—C2D—H2DA	110.9
C3B—C2B—H2BB	111.0	C3D—C2D—H2DB	110.9
C1B—C2B—H2BB	111.0	C1D—C2D—H2DB	110.9
H2BA—C2B—H2BB	109.0	H2DA—C2D—H2DB	108.9
C4B—C3B—C2B	118.14 (18)	C4D—C3D—C2D	119.16 (18)
C4B—C3B—C16B	115.15 (17)	C4D—C3D—C16D	115.05 (17)
C2B—C3B—C16B	104.61 (17)	C2D—C3D—C16D	104.44 (17)
C4B—C3B—H3BA	106.0	C4D—C3D—H3DA	105.7
C2B—C3B—H3BA	106.0	C2D—C3D—H3DA	105.7
C16B—C3B—H3BA	106.0	C16D—C3D—H3DA	105.7
C3B—C4B—C5B	110.24 (17)	C3D—C4D—C5D	110.77 (18)
C3B—C4B—C13B	108.67 (16)	C3D—C4D—C13D	109.29 (17)
C5B—C4B—C13B	110.65 (18)	C5D—C4D—C13D	110.39 (18)
C3B—C4B—H4BA	109.1	C3D—C4D—H4DA	108.8
C5B—C4B—H4BA	109.1	C5D—C4D—H4DA	108.8
C13B—C4B—H4BA	109.1	C13D—C4D—H4DA	108.8
C6B—C5B—C4B	113.50 (18)	C6D—C5D—C4D	113.58 (19)
C6B—C5B—H5BA	108.9	C6D—C5D—H5DA	108.8
C4B—C5B—H5BA	108.9	C4D—C5D—H5DA	108.8
C6B—C5B—H5BB	108.9	C6D—C5D—H5DB	108.8
C4B—C5B—H5BB	108.9	C4D—C5D—H5DB	108.8
H5BA—C5B—H5BB	107.7	H5DA—C5D—H5DB	107.7
C7B—C6B—C5B	124.8 (2)	C7D—C6D—C5D	125.5 (2)
C7B—C6B—H6BA	117.6	C7D—C6D—H6DA	117.2
C5B—C6B—H6BA	117.6	C5D—C6D—H6DA	117.2
C6B—C7B—C8B	119.8 (2)	C6D—C7D—C8D	120.7 (2)
C6B—C7B—C12B	123.3 (2)	C6D—C7D—C12D	122.7 (2)
C8B—C7B—C12B	116.9 (2)	C8D—C7D—C12D	116.57 (19)
C9B—C8B—C7B	110.19 (19)	C7D—C8D—C9D	110.84 (19)
C9B—C8B—H8BA	109.6	C7D—C8D—H8DA	109.5
C7B—C8B—H8BA	109.6	C9D—C8D—H8DA	109.5
C9B—C8B—H8BB	109.6	C7D—C8D—H8DB	109.5
C7B—C8B—H8BB	109.6	C9D—C8D—H8DB	109.5
H8BA—C8B—H8BB	108.1	H8DA—C8D—H8DB	108.1
C8B—C9B—C10B	110.5 (2)	C10D—C9D—C8D	110.2 (2)
C8B—C9B—Cl1B	110.04 (16)	C10D—C9D—Cl1D	110.44 (17)
C10B—C9B—Cl1B	110.70 (18)	C8D—C9D—Cl1D	110.18 (16)
C8B—C9B—H9BA	108.5	C10D—C9D—H9DA	108.7
C10B—C9B—H9BA	108.5	C8D—C9D—H9DA	108.7
Cl1B—C9B—H9BA	108.5	Cl1D—C9D—H9DA	108.7
C9B—C10B—C11B	108.99 (19)	C9D—C10D—C11D	109.25 (19)
C9B—C10B—H10C	109.9	C9D—C10D—H10G	109.8
C11B—C10B—H10C	109.9	C11D—C10D—H10G	109.8
C9B—C10B—H10D	109.9	C9D—C10D—H10H	109.8
C11B—C10B—H10D	109.9	C11D—C10D—H10H	109.8
H10C—C10B—H10D	108.3	H10G—C10D—H10H	108.3
C10B—C11B—C12B	113.73 (18)	C10D—C11D—C12D	114.48 (18)
C10B—C11B—H11C	108.8	C10D—C11D—H11G	108.6
C12B—C11B—H11C	108.8	C12D—C11D—H11G	108.6
C10B—C11B—H11D	108.8	C10D—C11D—H11H	108.6
C12B—C11B—H11D	108.8	C12D—C11D—H11H	108.6
H11C—C11B—H11D	107.7	H11G—C11D—H11H	107.6
C7B—C12B—C27B	109.01 (18)	C27D—C12D—C7D	108.83 (17)
C7B—C12B—C11B	108.81 (19)	C27D—C12D—C11D	109.63 (18)
C27B—C12B—C11B	109.11 (18)	C7D—C12D—C11D	107.66 (18)
C7B—C12B—C13B	110.03 (17)	C27D—C12D—C13D	111.79 (18)
C27B—C12B—C13B	111.64 (18)	C7D—C12D—C13D	110.16 (17)
C11B—C12B—C13B	108.19 (17)	C11D—C12D—C13D	108.70 (16)
C14B—C13B—C4B	110.83 (17)	C14D—C13D—C4D	110.23 (18)
C14B—C13B—C12B	113.57 (17)	C14D—C13D—C12D	113.51 (17)
C4B—C13B—C12B	112.72 (17)	C4D—C13D—C12D	113.38 (16)
C14B—C13B—H13B	106.4	C14D—C13D—H13D	106.4
C4B—C13B—H13B	106.4	C4D—C13D—H13D	106.4
C12B—C13B—H13B	106.4	C12D—C13D—H13D	106.4
C13B—C14B—C15B	114.46 (18)	C15D—C14D—C13D	114.56 (18)
C13B—C14B—H14C	108.6	C15D—C14D—H14G	108.6
C15B—C14B—H14C	108.6	C13D—C14D—H14G	108.6
C13B—C14B—H14D	108.6	C15D—C14D—H14H	108.6
C15B—C14B—H14D	108.6	C13D—C14D—H14H	108.6
H14C—C14B—H14D	107.6	H14G—C14D—H14H	107.6
C16B—C15B—C14B	111.93 (17)	C14D—C15D—C16D	112.09 (18)
C16B—C15B—H15C	109.2	C14D—C15D—H15G	109.2
C14B—C15B—H15C	109.2	C16D—C15D—H15G	109.2
C16B—C15B—H15D	109.2	C14D—C15D—H15H	109.2
C14B—C15B—H15D	109.2	C16D—C15D—H15H	109.2
H15C—C15B—H15D	107.9	H15G—C15D—H15H	107.9
C15B—C16B—C3B	106.10 (17)	C26D—C16D—C15D	111.23 (18)
C15B—C16B—C26B	111.03 (17)	C26D—C16D—C3D	112.32 (17)
C3B—C16B—C26B	112.64 (17)	C15D—C16D—C3D	105.71 (17)
C15B—C16B—C17B	116.67 (17)	C26D—C16D—C17D	110.21 (18)
C3B—C16B—C17B	99.88 (16)	C15D—C16D—C17D	116.34 (18)
C26B—C16B—C17B	110.03 (17)	C3D—C16D—C17D	100.51 (16)
C18B—C17B—C1B	111.84 (18)	C16D—C17D—C18D	120.10 (17)
C18B—C17B—C16B	119.60 (17)	C16D—C17D—C1D	103.57 (18)
C1B—C17B—C16B	103.19 (16)	C18D—C17D—C1D	111.20 (18)
C18B—C17B—H17B	107.2	C16D—C17D—H17D	107.1
C1B—C17B—H17B	107.2	C18D—C17D—H17D	107.1
C16B—C17B—H17B	107.2	C1D—C17D—H17D	107.1
C25B—C18B—C17B	113.19 (18)	C25D—C18D—C19D	110.56 (19)
C25B—C18B—C19B	110.20 (18)	C25D—C18D—C17D	112.59 (19)
C17B—C18B—C19B	109.46 (17)	C19D—C18D—C17D	109.77 (18)
C25B—C18B—H18B	107.9	C25D—C18D—H18D	107.9
C17B—C18B—H18B	107.9	C19D—C18D—H18D	107.9
C19B—C18B—H18B	107.9	C17D—C18D—H18D	107.9
C20B—C19B—C18B	114.39 (18)	C20D—C19D—C18D	115.01 (19)
C20B—C19B—H19C	108.7	C20D—C19D—H19G	108.5
C18B—C19B—H19C	108.7	C18D—C19D—H19G	108.5
C20B—C19B—H19D	108.7	C20D—C19D—H19H	108.5
C18B—C19B—H19D	108.7	C18D—C19D—H19H	108.5
H19C—C19B—H19D	107.6	H19G—C19D—H19H	107.5
C19B—C20B—C21B	113.07 (18)	C19D—C20D—C21D	112.3 (2)
C19B—C20B—H20C	109.0	C19D—C20D—H20G	109.1
C21B—C20B—H20C	109.0	C21D—C20D—H20G	109.1
C19B—C20B—H20D	109.0	C19D—C20D—H20H	109.1
C21B—C20B—H20D	109.0	C21D—C20D—H20H	109.1
H20C—C20B—H20D	107.8	H20G—C20D—H20H	107.9
C20B—C21B—C22B	114.92 (19)	C20D—C21D—C22D	115.8 (2)
C20B—C21B—H21C	108.5	C20D—C21D—H21G	108.3
C22B—C21B—H21C	108.5	C22D—C21D—H21G	108.3
C20B—C21B—H21D	108.5	C20D—C21D—H21H	108.3
C22B—C21B—H21D	108.5	C22D—C21D—H21H	108.3
H21C—C21B—H21D	107.5	H21G—C21D—H21H	107.4
C23B—C22B—C21B	113.0 (2)	C23D—C22D—C24D	109.7 (2)
C23B—C22B—C24B	110.3 (2)	C23D—C22D—C21D	112.8 (2)
C21B—C22B—C24B	110.0 (2)	C24D—C22D—C21D	110.6 (2)
C23B—C22B—H22B	107.8	C23D—C22D—H22D	107.9
C21B—C22B—H22B	107.8	C24D—C22D—H22D	107.9
C24B—C22B—H22B	107.8	C21D—C22D—H22D	107.9
C22B—C23B—H23D	109.5	C22D—C23D—H23J	109.5
C22B—C23B—H23E	109.5	C22D—C23D—H23K	109.5
H23D—C23B—H23E	109.5	H23J—C23D—H23K	109.5
C22B—C23B—H23F	109.5	C22D—C23D—H23L	109.5
H23D—C23B—H23F	109.5	H23J—C23D—H23L	109.5
H23E—C23B—H23F	109.5	H23K—C23D—H23L	109.5
C22B—C24B—H24D	109.5	C22D—C24D—H24J	109.5
C22B—C24B—H24E	109.5	C22D—C24D—H24K	109.5
H24D—C24B—H24E	109.5	H24J—C24D—H24K	109.5
C22B—C24B—H24F	109.5	C22D—C24D—H24L	109.5
H24D—C24B—H24F	109.5	H24J—C24D—H24L	109.5
H24E—C24B—H24F	109.5	H24K—C24D—H24L	109.5
C18B—C25B—H25D	109.5	C18D—C25D—H25J	109.5
C18B—C25B—H25E	109.5	C18D—C25D—H25K	109.5
H25D—C25B—H25E	109.5	H25J—C25D—H25K	109.5
C18B—C25B—H25F	109.5	C18D—C25D—H25L	109.5
H25D—C25B—H25F	109.5	H25J—C25D—H25L	109.5
H25E—C25B—H25F	109.5	H25K—C25D—H25L	109.5
C16B—C26B—H26D	109.5	C16D—C26D—H26J	109.5
C16B—C26B—H26E	109.5	C16D—C26D—H26K	109.5
H26D—C26B—H26E	109.5	H26J—C26D—H26K	109.5
C16B—C26B—H26F	109.5	C16D—C26D—H26L	109.5
H26D—C26B—H26F	109.5	H26J—C26D—H26L	109.5
H26E—C26B—H26F	109.5	H26K—C26D—H26L	109.5
C12B—C27B—H27D	109.5	C12D—C27D—H27J	109.5
C12B—C27B—H27E	109.5	C12D—C27D—H27K	109.5
H27D—C27B—H27E	109.5	H27J—C27D—H27K	109.5
C12B—C27B—H27F	109.5	C12D—C27D—H27L	109.5
H27D—C27B—H27F	109.5	H27J—C27D—H27L	109.5
H27E—C27B—H27F	109.5	H27K—C27D—H27L	109.5
			
C17A—C1A—C2A—C3A	10.2 (2)	C17C—C1C—C2C—C3C	10.3 (2)
C1A—C2A—C3A—C4A	−164.18 (18)	C1C—C2C—C3C—C4C	−166.15 (19)
C1A—C2A—C3A—C16A	−34.6 (2)	C1C—C2C—C3C—C16C	−36.2 (2)
C2A—C3A—C4A—C5A	−53.7 (3)	C2C—C3C—C4C—C5C	−57.0 (3)
C16A—C3A—C4A—C5A	−178.72 (18)	C16C—C3C—C4C—C5C	178.51 (18)
C2A—C3A—C4A—C13A	−175.31 (19)	C2C—C3C—C4C—C13C	−178.34 (19)
C16A—C3A—C4A—C13A	59.7 (2)	C16C—C3C—C4C—C13C	57.1 (2)
C3A—C4A—C5A—C6A	−161.50 (18)	C3C—C4C—C5C—C6C	−160.24 (18)
C13A—C4A—C5A—C6A	−40.9 (2)	C13C—C4C—C5C—C6C	−39.0 (3)
C4A—C5A—C6A—C7A	11.8 (3)	C4C—C5C—C6C—C7C	9.8 (3)
C5A—C6A—C7A—C8A	−177.9 (2)	C5C—C6C—C7C—C8C	−177.8 (2)
C5A—C6A—C7A—C12A	1.8 (4)	C5C—C6C—C7C—C12C	0.6 (4)
C6A—C7A—C8A—C9A	−128.5 (2)	C6C—C7C—C8C—C9C	−131.3 (2)
C12A—C7A—C8A—C9A	51.9 (3)	C12C—C7C—C8C—C9C	50.3 (3)
C7A—C8A—C9A—C10A	−56.4 (3)	C7C—C8C—C9C—C10C	−56.9 (3)
C7A—C8A—C9A—Cl1A	−178.29 (16)	C7C—C8C—C9C—Cl1C	−178.88 (17)
C8A—C9A—C10A—C11A	60.2 (3)	C8C—C9C—C10C—C11C	60.8 (2)
Cl1A—C9A—C10A—C11A	−178.25 (15)	Cl1C—C9C—C10C—C11C	−177.27 (15)
C9A—C10A—C11A—C12A	−58.9 (3)	C9C—C10C—C11C—C12C	−57.9 (3)
C6A—C7A—C12A—C27A	−108.3 (2)	C6C—C7C—C12C—C27C	−104.2 (2)
C8A—C7A—C12A—C27A	71.4 (2)	C8C—C7C—C12C—C27C	74.1 (2)
C6A—C7A—C12A—C11A	132.5 (2)	C6C—C7C—C12C—C11C	137.0 (2)
C8A—C7A—C12A—C11A	−47.9 (3)	C8C—C7C—C12C—C11C	−44.6 (2)
C6A—C7A—C12A—C13A	14.7 (3)	C6C—C7C—C12C—C13C	19.0 (3)
C8A—C7A—C12A—C13A	−165.61 (18)	C8C—C7C—C12C—C13C	−162.69 (18)
C10A—C11A—C12A—C7A	51.2 (3)	C10C—C11C—C12C—C7C	48.3 (2)
C10A—C11A—C12A—C27A	−67.7 (2)	C10C—C11C—C12C—C27C	−70.0 (2)
C10A—C11A—C12A—C13A	170.38 (18)	C10C—C11C—C12C—C13C	167.21 (18)
C3A—C4A—C13A—C14A	−50.6 (2)	C3C—C4C—C13C—C14C	−49.7 (2)
C5A—C4A—C13A—C14A	−172.34 (18)	C5C—C4C—C13C—C14C	−171.70 (18)
C3A—C4A—C13A—C12A	−179.41 (17)	C3C—C4C—C13C—C12C	−177.77 (17)
C5A—C4A—C13A—C12A	58.9 (2)	C5C—C4C—C13C—C12C	60.2 (2)
C7A—C12A—C13A—C14A	−172.32 (18)	C7C—C12C—C13C—C4C	−49.1 (2)
C27A—C12A—C13A—C14A	−50.8 (2)	C27C—C12C—C13C—C4C	71.8 (2)
C11A—C12A—C13A—C14A	69.9 (2)	C11C—C12C—C13C—C4C	−167.18 (18)
C7A—C12A—C13A—C4A	−44.5 (2)	C7C—C12C—C13C—C14C	−176.14 (18)
C27A—C12A—C13A—C4A	77.0 (2)	C27C—C12C—C13C—C14C	−55.2 (2)
C11A—C12A—C13A—C4A	−162.32 (18)	C11C—C12C—C13C—C14C	65.8 (2)
C4A—C13A—C14A—C15A	50.0 (3)	C4C—C13C—C14C—C15C	51.1 (2)
C12A—C13A—C14A—C15A	178.32 (18)	C12C—C13C—C14C—C15C	178.63 (18)
C13A—C14A—C15A—C16A	−53.9 (3)	C13C—C14C—C15C—C16C	−55.6 (2)
C4A—C3A—C16A—C15A	−61.8 (2)	C4C—C3C—C16C—C15C	−59.5 (2)
C2A—C3A—C16A—C15A	166.44 (17)	C2C—C3C—C16C—C15C	169.23 (17)
C4A—C3A—C16A—C26A	59.8 (2)	C4C—C3C—C16C—C26C	62.1 (2)
C2A—C3A—C16A—C26A	−72.0 (2)	C2C—C3C—C16C—C26C	−69.1 (2)
C4A—C3A—C16A—C17A	176.89 (17)	C4C—C3C—C16C—C17C	178.82 (17)
C2A—C3A—C16A—C17A	45.08 (19)	C2C—C3C—C16C—C17C	47.6 (2)
C14A—C15A—C16A—C3A	56.2 (2)	C14C—C15C—C16C—C3C	56.3 (2)
C14A—C15A—C16A—C26A	−66.0 (2)	C14C—C15C—C16C—C26C	−66.8 (2)
C14A—C15A—C16A—C17A	167.06 (18)	C14C—C15C—C16C—C17C	166.51 (18)
C2A—C1A—C17A—C18A	146.80 (18)	C3C—C16C—C17C—C18C	−164.51 (19)
C2A—C1A—C17A—C16A	17.0 (2)	C15C—C16C—C17C—C18C	81.5 (3)
C3A—C16A—C17A—C18A	−162.45 (18)	C26C—C16C—C17C—C18C	−45.6 (3)
C15A—C16A—C17A—C18A	83.6 (2)	C3C—C16C—C17C—C1C	−39.6 (2)
C26A—C16A—C17A—C18A	−43.9 (2)	C15C—C16C—C17C—C1C	−153.53 (19)
C3A—C16A—C17A—C1A	−37.17 (19)	C26C—C16C—C17C—C1C	79.3 (2)
C15A—C16A—C17A—C1A	−151.07 (18)	C2C—C1C—C17C—C18C	149.04 (19)
C26A—C16A—C17A—C1A	81.4 (2)	C2C—C1C—C17C—C16C	18.5 (2)
C1A—C17A—C18A—C25A	−176.97 (18)	C16C—C17C—C18C—C25C	−54.5 (3)
C16A—C17A—C18A—C25A	−55.9 (3)	C1C—C17C—C18C—C25C	−175.36 (18)
C1A—C17A—C18A—C19A	59.3 (2)	C16C—C17C—C18C—C19C	−176.86 (19)
C16A—C17A—C18A—C19A	−179.65 (18)	C1C—C17C—C18C—C19C	62.2 (2)
C25A—C18A—C19A—C20A	71.2 (2)	C25C—C18C—C19C—C20C	76.0 (3)
C17A—C18A—C19A—C20A	−163.32 (19)	C17C—C18C—C19C—C20C	−159.2 (2)
C18A—C19A—C20A—C21A	177.8 (2)	C18C—C19C—C20C—C21C	175.5 (2)
C19A—C20A—C21A—C22A	−171.3 (2)	C19C—C20C—C21C—C22C	−168.0 (3)
C20A—C21A—C22A—C23A	−62.3 (3)	C20C—C21C—C22C—C23C	−62.7 (4)
C20A—C21A—C22A—C24A	173.8 (2)	C20C—C21C—C22C—C24C	174.6 (3)
C17B—C1B—C2B—C3B	8.2 (2)	C17D—C1D—C2D—C3D	9.1 (2)
C1B—C2B—C3B—C4B	−163.73 (18)	C1D—C2D—C3D—C4D	−164.13 (19)
C1B—C2B—C3B—C16B	−34.1 (2)	C1D—C2D—C3D—C16D	−34.0 (2)
C2B—C3B—C4B—C5B	−54.0 (3)	C2D—C3D—C4D—C5D	−52.7 (3)
C16B—C3B—C4B—C5B	−178.54 (18)	C16D—C3D—C4D—C5D	−177.83 (18)
C2B—C3B—C4B—C13B	−175.40 (18)	C2D—C3D—C4D—C13D	−174.50 (19)
C16B—C3B—C4B—C13B	60.0 (2)	C16D—C3D—C4D—C13D	60.3 (2)
C3B—C4B—C5B—C6B	−160.22 (18)	C3D—C4D—C5D—C6D	−161.27 (18)
C13B—C4B—C5B—C6B	−40.0 (3)	C13D—C4D—C5D—C6D	−40.1 (2)
C4B—C5B—C6B—C7B	11.9 (3)	C4D—C5D—C6D—C7D	12.8 (3)
C5B—C6B—C7B—C8B	−179.2 (2)	C5D—C6D—C7D—C8D	−178.8 (2)
C5B—C6B—C7B—C12B	0.3 (4)	C5D—C6D—C7D—C12D	−0.1 (4)
C6B—C7B—C8B—C9B	−128.7 (3)	C6D—C7D—C8D—C9D	−128.9 (2)
C12B—C7B—C8B—C9B	51.8 (3)	C12D—C7D—C8D—C9D	52.3 (3)
C7B—C8B—C9B—C10B	−58.6 (3)	C7D—C8D—C9D—C10D	−57.1 (3)
C7B—C8B—C9B—Cl1B	178.89 (17)	C7D—C8D—C9D—Cl1D	−179.25 (16)
C8B—C9B—C10B—C11B	62.1 (3)	C8D—C9D—C10D—C11D	60.3 (3)
Cl1B—C9B—C10B—C11B	−175.74 (16)	Cl1D—C9D—C10D—C11D	−177.68 (15)
C9B—C10B—C11B—C12B	−57.9 (3)	C9D—C10D—C11D—C12D	−58.8 (3)
C6B—C7B—C12B—C27B	−106.3 (3)	C6D—C7D—C12D—C27D	−107.2 (2)
C8B—C7B—C12B—C27B	73.2 (2)	C8D—C7D—C12D—C27D	71.6 (2)
C6B—C7B—C12B—C11B	134.8 (2)	C6D—C7D—C12D—C11D	134.0 (2)
C8B—C7B—C12B—C11B	−45.7 (3)	C8D—C7D—C12D—C11D	−47.2 (2)
C6B—C7B—C12B—C13B	16.4 (3)	C6D—C7D—C12D—C13D	15.7 (3)
C8B—C7B—C12B—C13B	−164.08 (19)	C8D—C7D—C12D—C13D	−165.55 (18)
C10B—C11B—C12B—C7B	48.3 (3)	C10D—C11D—C12D—C27D	−68.0 (2)
C10B—C11B—C12B—C27B	−70.6 (3)	C10D—C11D—C12D—C7D	50.2 (2)
C10B—C11B—C12B—C13B	167.8 (2)	C10D—C11D—C12D—C13D	169.55 (18)
C3B—C4B—C13B—C14B	−52.0 (2)	C3D—C4D—C13D—C14D	−51.8 (2)
C5B—C4B—C13B—C14B	−173.15 (17)	C5D—C4D—C13D—C14D	−173.91 (18)
C3B—C4B—C13B—C12B	179.48 (18)	C3D—C4D—C13D—C12D	179.66 (17)
C5B—C4B—C13B—C12B	58.3 (2)	C5D—C4D—C13D—C12D	57.6 (2)
C7B—C12B—C13B—C14B	−172.34 (18)	C27D—C12D—C13D—C14D	−49.9 (2)
C27B—C12B—C13B—C14B	−51.2 (2)	C7D—C12D—C13D—C14D	−171.02 (18)
C11B—C12B—C13B—C14B	68.9 (2)	C11D—C12D—C13D—C14D	71.3 (2)
C7B—C12B—C13B—C4B	−45.2 (2)	C27D—C12D—C13D—C4D	76.9 (2)
C27B—C12B—C13B—C4B	75.9 (2)	C7D—C12D—C13D—C4D	−44.2 (2)
C11B—C12B—C13B—C4B	−163.99 (18)	C11D—C12D—C13D—C4D	−161.95 (18)
C4B—C13B—C14B—C15B	51.4 (2)	C4D—C13D—C14D—C15D	51.1 (2)
C12B—C13B—C14B—C15B	179.45 (18)	C12D—C13D—C14D—C15D	179.57 (18)
C13B—C14B—C15B—C16B	−53.9 (3)	C13D—C14D—C15D—C16D	−54.3 (3)
C14B—C15B—C16B—C3B	54.5 (2)	C14D—C15D—C16D—C26D	−67.3 (2)
C14B—C15B—C16B—C26B	−68.1 (2)	C14D—C15D—C16D—C3D	54.8 (2)
C14B—C15B—C16B—C17B	164.71 (18)	C14D—C15D—C16D—C17D	165.36 (18)
C4B—C3B—C16B—C15B	−60.6 (2)	C4D—C3D—C16D—C26D	60.9 (2)
C2B—C3B—C16B—C15B	168.00 (17)	C2D—C3D—C16D—C26D	−71.6 (2)
C4B—C3B—C16B—C26B	61.0 (2)	C4D—C3D—C16D—C15D	−60.6 (2)
C2B—C3B—C16B—C26B	−70.3 (2)	C2D—C3D—C16D—C15D	166.91 (18)
C4B—C3B—C16B—C17B	177.74 (17)	C4D—C3D—C16D—C17D	178.03 (18)
C2B—C3B—C16B—C17B	46.37 (19)	C2D—C3D—C16D—C17D	45.5 (2)
C2B—C1B—C17B—C18B	149.97 (18)	C26D—C16D—C17D—C18D	−44.7 (3)
C2B—C1B—C17B—C16B	20.1 (2)	C15D—C16D—C17D—C18D	83.1 (2)
C15B—C16B—C17B—C18B	81.5 (2)	C3D—C16D—C17D—C18D	−163.34 (19)
C3B—C16B—C17B—C18B	−164.77 (18)	C26D—C16D—C17D—C1D	80.1 (2)
C26B—C16B—C17B—C18B	−46.1 (2)	C15D—C16D—C17D—C1D	−152.13 (19)
C15B—C16B—C17B—C1B	−153.57 (19)	C3D—C16D—C17D—C1D	−38.6 (2)
C3B—C16B—C17B—C1B	−39.8 (2)	C2D—C1D—C17D—C16D	18.8 (2)
C26B—C16B—C17B—C1B	78.8 (2)	C2D—C1D—C17D—C18D	149.12 (19)
C1B—C17B—C18B—C25B	−174.70 (18)	C16D—C17D—C18D—C25D	−54.4 (3)
C16B—C17B—C18B—C25B	−54.0 (3)	C1D—C17D—C18D—C25D	−175.40 (19)
C1B—C17B—C18B—C19B	62.0 (2)	C16D—C17D—C18D—C19D	−177.99 (19)
C16B—C17B—C18B—C19B	−177.35 (18)	C1D—C17D—C18D—C19D	61.0 (2)
C25B—C18B—C19B—C20B	72.0 (2)	C25D—C18D—C19D—C20D	70.6 (3)
C17B—C18B—C19B—C20B	−162.91 (19)	C17D—C18D—C19D—C20D	−164.6 (2)
C18B—C19B—C20B—C21B	175.22 (19)	C18D—C19D—C20D—C21D	175.2 (2)
C19B—C20B—C21B—C22B	−173.3 (2)	C19D—C20D—C21D—C22D	−174.2 (2)
C20B—C21B—C22B—C23B	−66.5 (3)	C20D—C21D—C22D—C23D	−67.7 (3)
C20B—C21B—C22B—C24B	169.7 (2)	C20D—C21D—C22D—C24D	169.0 (2)
